# A taxonomic review on the species of *Tetraserica* Ahrens, 2004, of China (Coleoptera, Scarabaeidae, Sericini)

**DOI:** 10.3897/zookeys.448.8429

**Published:** 2014-10-20

**Authors:** Wan-Gang Liu, Silvia Fabrizi, Ming Bai, Xing-Ke Yang, Dirk Ahrens

**Affiliations:** 1 Key Laboratory of Zoological Systematics and Evolution, Institute of Zoology, Chinese Academy of Sciences, Box 92, No. 1, Beichen West Road, Chaoyang District, Beijing, 100101, P.R. China; 2 University of Chinese Academy of Sciences, Yuquan Road, Shijingshan, Beijing, 100039, P.R. China; 3 Zoologisches Forschungsmuseum A. Koenig, Adenauerallee 160, 53113 Bonn, Germany

**Keywords:** Beetles, chafers, *Tetraserica*, China, new species

## Abstract

A review on the Chinese species of *Tetraserica* Ahrens, 2004, is presented. The lectotype of *Tetraserica tonkinensis* (Moser, 1908), **comb. n.** is designated. Twenty-nine new *Tetraserica* species are described from China and adjacent regions: *Tetraserica anhuaensis* **sp. n.**, *Tetraserica changjiangensis* **sp. n.**, *Tetraserica changshouensis* **sp. n.**, *Tetraserica damaidiensis* **sp. n.**, *Tetraserica daqingshanica* **sp. n.**, *Tetraserica fikaceki* **sp. n.**, *Tetraserica graciliforceps* **sp. n.**, *Tetraserica jinghongensis* **sp. n.**, *Tetraserica leishanica* **sp. n.**, *Tetraserica liangheensis* **sp. n.**, *Tetraserica linaoshanica* **sp. n.**, *Tetraserica longipenis* **sp. n.**, *Tetraserica longzhouensis* **sp. n.**, *Tetraserica maoershanensis* **sp. n.**, *Tetraserica mengeana* **sp. n.**, *Tetraserica menglongensis* **sp. n.**, *Tetraserica pingjiangensis* **sp. n.**, *Tetraserica ruiliana* **sp. n.**, *Tetraserica ruiliensis* **sp. n.**, *Tetraserica sculptilis* **sp. n.**, *Tetraserica shangsiensis* **sp. n.**, *Tetraserica shunbiensis* **sp. n.**, *Tetraserica sigulianshanica* **sp. n.**, *Tetraserica tianchiensis* **sp. n.**, *Tetraserica wandingensis* **sp. n.**, *Tetraserica wangtongensis* **sp. n.**, *Tetraserica xichouensis* **sp. n.**, *Tetraserica yaoanica* **sp. n.**, *Tetraserica yaoquensis* **sp. n.** A key to the Chinese *Tetraserica* species is given, species distribution as well as the habitus and male genitalia of all species are illustrated.

## Introduction

The genus *Tetraserica* was established by Ahrens (2004). The genus included so far 8 nominal species from Indian subcontinent and Myanmar. Other species are known or described from India, Indochina, Philippines, Sumatra and Borneo, but they are not yet formally transferred to *Tetraserica*. Based on the results of this study, the genus is for the first time recorded for China. Recent molecular work confirmed the monophyly of *Tetraserica* (Ahrens and Vogler 2008; Liu et al., unpublished data).

In this study, we examined the material collected in China mainland and deposited in Chinese institutional collections as well as various European and American collections. We found twenty-nine new taxa, which are described herein. Additionally, non-Chinese records are added to the species recorded from China, while the taxa occurring exclusively out of China are not revised herein. A key to the Chinese *Tetraserica* species is given, species distribution, as well as habitus and male genitalia, are illustrated.

## Material and methods

The terminology and methods used for measurements, specimen dissection and genital preparation follow Ahrens (2004). Data from specimens examined are cited in the text with original label contents given in quotation marks, multiple labels are separated by a “/”. Male genitalia were glued to a small pointed card attached to the specimen. Descriptions and illustrations of new taxa are based on the holotype or lectotype specimen, while the variation of other specimens is given separately. All descriptions and measurements were made under an Olympus SZX 12 microscope, and all genital and habitus illustrations were made with a digital camera (AxioCam HRc) attached to a stereo microscope (Zeiss Stereo Discovery V20) and Axio Version 4.8 software. The distribution maps were generated using Q-GIS 2.0.1 and Inscape software.

Type specimens and other examined material are deposited in the following institutions or collections:

BPBM Bernice P. Bishop Museum, Honolulu (Hawai), U.S.A.;

CAU Department of Entomology, China Agricultural University, Beijing, China;

CNAR Collection A. Napolov, Riga, Latvia;

CPPB Coll. Petr Pacholátko, Brno, Czech Republic;

HBUM Museum of Hebei University, Baoding (Hebei Prov.), China;

IZAS Institute of Zoology, Chinese Academy of Sciences, Beijing, China;

MZUF Museo Zoologico „La Specola”, Università di Firenze, Italy;

NHMW Natural History Museum Vienna, Austria;

NME Naturkundemuseum Erfurt, Germany;

NMPC National Museum Prague (Natural History), Czech Republic;

NUYS Northwest A & F University, Yangling (Shaanxi Prov.), China;

SYUG Sun Yat-Sen University, Guangzhou (Guangdong Prov.), China;

USNM National Museum of Natural History, Washington D.C., U.S.A.;

ZFMK Zoologisches Forschungsmuseum A. Koenig, Bonn, Germany.

## Taxonomy

### Tetraserica

Taxon classificationAnimaliaColeopteraScarabaeidae

Ahrens, 2004

Tetraserica Ahrens, 2004: 168 (type species by original designation: *Neoserica gestroi* Brenske, 1898).

#### Diagnosis.

Body moderately large to large (6–12 mm), mostly dark brown; ventral surface reddish brown; dorsal surface dull and glabrous.

Labroclypeus subtrapezoidal, wider than long, widest at base, lateral margins moderately convex and convergent to strongly rounded anterior angles, anterior margin weakly sinuate medially, margins moderately reflexed; surface weakly convex, moderately shiny, finely and densely punctate; frontoclypeal suture indistinctly incised, flat and weakly curved medially; ocular canthus short and triangular, impunctate, with a single terminal seta. Frons dull, with sparse, fine punctures, with single erect setae beside each eye. Antenna yellowish, with 10 antennomeres; club composed of 4 antennomeres in male, straight, rarely longer than 1.5 times as the remaining antennomeres combined; club in female composed of 3 antennomeres, as long as the remaining antennomeres combined. Mentum elevated and slightly flattened anteriorly.

Pronotum moderately wide and strongly convex, lateral margins evenly convex, more strongly narrowed anteriorly towards sharp and slightly produced anterior angles. Anterior margin of pronotum slightly convex, with fine complete marginal line. Posterior angles blunt or strongly rounded. Surface finely and densely punctate, except minute setae glabrous, lateral and lateral anterior margins sparsely setose. Hypomeron not carinate. Scutellum triangular, finely and densely punctate.

Elytra oblong, widest just behind middle, striae distinctly impressed, finely and moderately densely punctate, intervals distinctly convex, with coarse and dense punctures concentrated along striae, with very minute setae in punctures; epipleural edge robust, ending at weakly curved and slightly blunt external apical angle of elytra, epipleura densely setose, apical border with a broad fringe of microtrichomes (100×).

Ventral surface weakly shiny, finely and densely punctate, metasternum sparsely covered with fine, short, or very minute setae, metacoxa glabrous, with a few single setae laterally; abdominal sternites finely and densely punctuate, with a transverse row of coarse punctures, each bearing a robust seta. Mesosternum between mesocoxae as wide as mesofemur. Pygidium weakly convex and dull, densely punctate, without smooth midline, almost glabrous, but with a few longer setae along apical margin; pygidium without strong sexual dimorphism.

Legs moderately wide; femora finely and sparsely punctate; metafemur wide and moderately shiny or dull, anterior margin acute, posterior margin smooth ventrally and only weakly widened in apical half, posterior margin smooth dorsally, with a few short setae basally. Metatibia moderately wide to wide and moderately long, widest at half of metatibial length, dorsal margin sharply carinate, with two groups of spines; lateral face finely and sparsely punctate; ventral edge finely serrated, with four robust equidistant setae, medial face smooth, apex interiorly near tarsal articulation with a shallow sinuation. Tarsomeres with fine, very dense setae ventrally on distal half, neither laterally nor dorsally carinate, dorsally smooth; metatarsomeres with a strongly serrated ridge ventrally and glabrous; first metatarsomere slightly shorter than two following tarsomeres combined, one third of its length longer than dorsal tibial spine. Protibia short, bidentate; anterior claws symmetrical, basal tooth of both claws bluntly truncate at apex.

Aedoeagus. Phallobasis with a more or less long median ventral extension.

#### Remarks.

So far seven species from Himalaya and the type species from Myanmar have been formally assigned to *Tetraserica* (Ahrens 2004; Ahrens and Fabrizi 2009). Most other oriental species (so far grouped with ‘*Neoserica*’) await taxonomic revision. *Tetraserica* differs from closely related genera *Microserica* Brenske, 1894, and *Trioserica* Moser, 1922, by the lacking ventral carina of hypomeron. From *Microserica* it also differs by the lacking sexual dimorphism of the pygidium, from *Trioserica* by the bidentate protibia. In contrast to the *Microserica*, species of *Tetraserica* are active at night and are attracted by light.

#### Distribution.

The genus is distributed almost in the entire Oriental region; we know described species so far assigned to “*Neoserica*” from Philippines, Indochina, Sumatra, and Borneo (Ahrens 2004). Except in Meghalaya and Himalaya, the genus does not occur on Indian subcontinent south of the Ganges.

#### Key to the Chinese species of *Tetraserica* (♂♂)

**Table:** 

1	Labroclypeus completely glabrous. Basal group of dorsal spines of metatibia before middle. Ratio ocular diameter/interocular distance <0.75	**4**
–	Labroclypeus with few fine setae. Basal group of dorsal spines of metatibia behind middle. Anterior margin of metafemur with continuously serrated adjacent line. Ratio ocular diameter/interocular distance >0.8	**2**
2	Metatibia more robust, ratio length/width: < 3.3. Metacoxa shorter, ratio length of metepisternum/metacoxa: 1/1.5	***Tetraserica anhuaensis* sp. n.**
–	Metatibia more slender, ratio length/width: >3.6. Metacoxa longer, ratio length of metepisternum/metacoxa: 1/1.68	**3**
3	Left paramere with long interior basal lobe (its length almost half of paramere length)	***Tetraserica yaoanica* sp. n.**
–	Left paramere with short interior basal lobe (its length less than quarter of paramere length)	***Tetraserica leishanica* sp. n.**
4	Posterior margin of metafemur straight or slightly convex	**10**
–	Posterior margin of metafemur with blunt tooth or sharp hook	**5**
5	Posterior margin of metafemur with blunt tooth	**6**
–	Posterior margin of metafemur with sharp hook	**9**
6	Left paramere long and narrow	***Tetraserica pingjiangensis* sp. n.**
–	Left paramere short and strout	**7**
7	Dorsal lobe of right paramere short, not exceeding length of ventral one	***Tetraserica maoershanensis* sp. n.**
–	Dorsal lobe of right paramere longer, exceeding length of ventral one	**8**
8	Left paramere more narrow, dorsal margin weakly and evenly curved	***Tetraserica sculptilis* sp. n.**
–	Left paramere stout, dorsal margin bluntly angulate	***Tetraserica daqingshanica* sp. n.**
9	Eyes smaller, ratio diameter/interocular distance: 0.59. Dorsal lobe of right paramere large and directed distally, exceeding ventral lobe by far	***Tetraserica liangheensis* sp. n.**
–	Eyes larger, ratio diameter/interocular distance: 0.72. Dorsal lobe of right paramere very small and bent basally	***Tetraserica wandingensis* sp. n.**
10	Ventral process of phallobasis short, distinctly shorter than phallobasis	**11**
–	Ventral process of phallobasis long, subequal to length of phallobasis	**19**
11	Ventral process of phallobasis short, subequal to at maximum half of length of phallobasis	**13**
–	Ventral process of phallobasis medium in length, about three quarter of phallobasis length	**12**
12	Metatibia more slender, ratio length/width ca. 3.4. Right paramere lacking basal lobe	***Tetraserica shunbiensis* sp. n.**
–	Metatibia more robust, ratio length/width ca. 3.0. Right paramere with long filiform and curved basal lobe	***Tetraserica sigulianshanica* sp. n.**
13	Eyes of medium size, ratio diameter/interocular distance ≥ 0.6	**15**
–	Eyes small, ratio diameter/interocular distance ≤ 0.5	**14**
14	Phallobasis in dorsal view only slightly asymmetric. Left and right parameres simple, without two lobes. Posterior angles of pronotum strongly rounded	***Tetraserica graciliforceps* sp. n.**
–	Phallobasis in dorsal view strongly asymmetric. Right paramere simple, left paramere with ventral lobe shorter than dorsal one. Posterior angles of pronotum moderately rounded	***Tetraserica longzhouensis* sp. n.**
15	Both parameres with dorsal and ventral lobe	***Tetraserica fikaceki* sp. n.**
–	One or both parameres simple, without two lobes	**16**
16	Both parameres simple, without two lobes	***Tetraserica damaidiensis* sp. n.**
–	One of parameres complex, with two lobes	**17**
17	Right paramere simple, left one with two lobes; right paramere basiventrally strongly widened towards apex	***Tetraserica yaoquensis* sp. n.**
–	Left paramere simple, right one with two lobes	**18**
18	Left paramere more slender, strongly evenly bent externally	***Tetraserica changjiangensis* sp. n.**
–	Left paramere more stout, almost straight	***Tetraserica wangtongensis* sp. n.**
19	Right paramere basally with brush of robust trichome-like spines	**20**
–	Right paramere without brush of spines	**25**
20	Left paramere composed of two lobes. Ventral lobe of right paramere abruptly and strongly widened at apex	***Tetraserica changshouensis* sp. n.**
–	Left paramere simple	**21**
21	Left paramere with small lateral basal tooth	***Tetraserica linaoshanica* sp. n.**
–	Left paramere without small lateral basal tooth	**22**
22	Left paramere split in two filiform branches behind middle	***Tetraserica mengeana* sp. n.**
–	Left paramere simply filiform	**23**
23	Left paramere bent twice	**24**
–	Left paramere evenly curved, without being clearly bent, before apex with tiny lateral tooth	***Tetraserica shangsiensis* sp. n.**
24	Dorsal lobe of right paramere very small	***Tetraserica xichouensis* sp. n.**
–	Dorsal lobe of right paramere large, nearly as long as ventral lobe	***Tetraserica tonkinensis* (Moser)**
25	Right paramere simple, not composed of two lobes	**26**
–	Right paramere composed of two lobes	**27**
26	Left paramere simple	***Tetraserica ruiliensis* sp. n.**
–	Left paramere composed of two lobes	***Tetraserica longipenis* sp. n.**
27	Left paramere simple	**28**
–	Left paramere composed of two lobes	**29**
28	Dorsal lobe of right paramere wide, with sickle-shaped, large apical hook	***Tetraserica tianchiensis* sp. n.**
–	Dorsal lobe of right paramere narrow, evenly curved and sharply pointed	***Tetraserica jinghongensis* sp. n.**
29	Dorsal lobe of right paramere triangular and short, sharply pointed	***Tetraserica menglongensis* sp. n.**
–	Dorsal lobe of right paramere convexly widened and elongate	***Tetraserica ruiliana* sp. n.**

### Tetraserica daqingshanica sp. n.

Taxon classificationAnimaliaColeopteraScarabaeidae

http://zoobank.org/74654444-A9CA-4349-8BD1-EE5C52AD9530

#### Type material examined.

Holotype: ♂ [China] “Mt. Daqingshan, Longzhou, Guangxi, 24.IV.1963, 600–700m, leg. Shi Yongshan” (IZAS). Paratype: 1 ♂ [China] “Mt. Daqingshan, Longzhou, Guangxi, 13.IV.1963, 360m, leg. Wang Shuyong” (ZFMK).

#### Description.

Body length: 9.2 mm, length of elytra: 7 mm, width: 5.5 mm.

Surface of labroclypeus and disc of frons glabrous. Smooth area anterior to eye twice as wide as long. Antennal club 1.1 times as long as remaining antennomeres combined. Eyes small; ratio of diameter/interocular width: 0.53. Ratio of length of metepisternum/metacoxa: 1/1.63. Metafemur dull, anterior margin acute, without submarginal serrated line; anterior row of setae-bearing punctures absent; posterior margin with a blunt tooth. Metatibia short and wide, ratio width/length: 1/3.1; basal group of dorsal spines of metatibia at first third of metatibial length.

**Figure 1.**
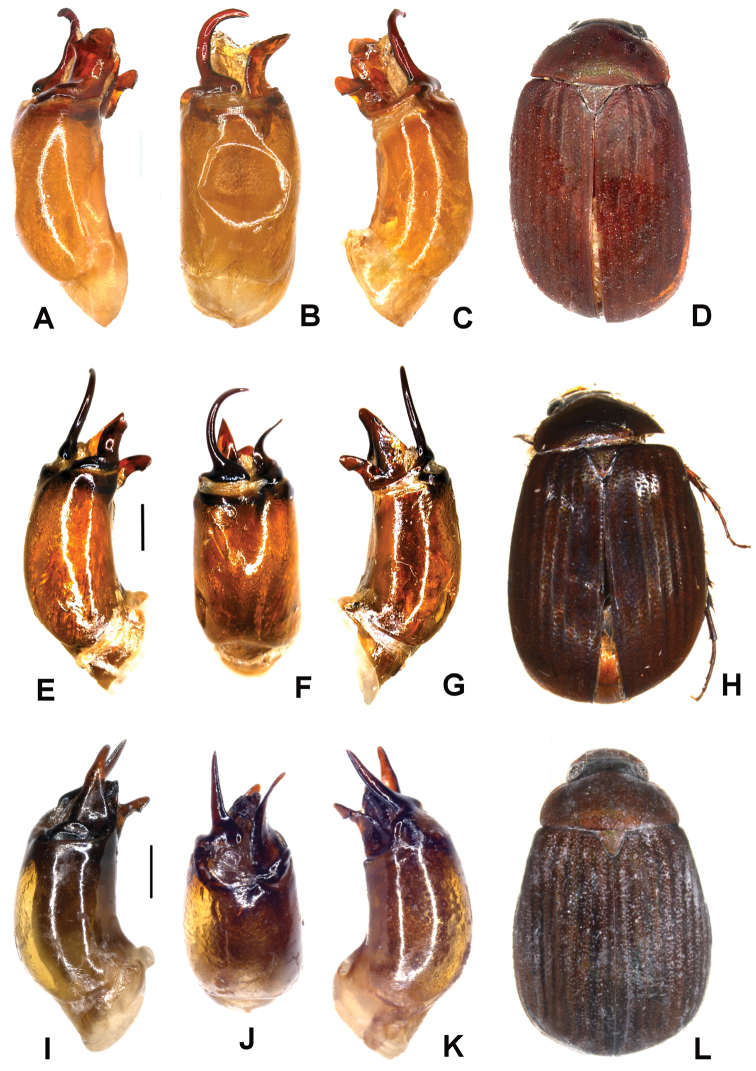
**A–D** *Tetraserica daqingshanica* sp. n. (holotype) **E–H** *Tetraserica sculptilis* Ahrens sp. n. (holotype) **I–L** *Tetraserica wangtongensis* sp. n. (holotype). **A, E, I** aedeagus, left side lateral view **C, G, K** aedeagus, right side lateral view **B, F, J** parameres, dorsal view **D, H, L** habitus (not to scale). Scale: 0.5 mm.

Aedeagus. Fig. 1A–C. Habitus: Fig. 1D.

Female unknown.

#### Variation.

Body length: 9.2 mm, length of elytra: 7.0–7.3 mm, width: 5.5–5.9 mm.

#### Diagnosis.

The new species differs from all other so far known *Tetraserica* species by the blunt tooth at the posterior margin of the metafemur.

#### Etymology.

The new species is named after the type locality, Mt. Daqingshan.

### Tetraserica sculptilis sp. n.

Taxon classificationAnimaliaColeopteraScarabaeidae

http://zoobank.org/1DD74C0C-602F-4EFC-970E-8DE9A3A25400

#### Type material examined.

Holotype: ♂ “China: Hubei; Dahongshan 1700m, Shuizhou VI-2003 leg. Ying et al.” (ZFMK). Paratypes. 1 ♂ [China] “Hekou, Southeast of Yunnan, 9.VI.1956, 1200m, leg. Panfilov” (IZAS), 1 ♂ [China] “Luxi, Yunnan, 22.V.1980, leg. Li Hongxing” (IZAS).

#### Description.

Body length: 9.1 mm, length of elytra: 7.3 mm, width: 5.4 mm.

Surface of labroclypeus and disc of frons glabrous. Smooth area anterior to eye twice as wide as long. Eyes moderately large; ratio of diameter/interocular width: 0.6. Antennal club 1.2 times as long as remaining antennomeres combined. Ratio of length of metepisternum/metacoxa: 1/1.7. Metafemur dull, anterior margin acute, without submarginal serrated line; anterior row of setae-bearing punctures absent; posterior margin with a blunt tooth. Metatibia short and wide, ratio width/length: 1/3.2; basal group of dorsal spines of metatibia at first third of metatibial length.

Aedeagus. Fig. 1E–G. Habitus: Fig. 1H.

Female unknown.

#### Variation.

Body length: 9.1–9.8 mm, length of elytra: 7.3–7.4 mm, width: 5.4–6.0 mm.

#### Diagnosis.

*Tetraserica sculptilis* sp. n. is in the external shape and morphology of the male genitalia very similar to *Tetraserica daqingshanica*. It differs only in the shape of the parameres: the left paramere is more narrow in *Tetraserica sculptilis* sp. n., its dorsal margin weakly and evenly curved (Fig. 1E).

#### Etymology.

From the Latin word *sculptilis* – modelled, sculptured, with reference to the shape of the aedeagus.

### Tetraserica wangtongensis sp. n.

Taxon classificationAnimaliaColeopteraScarabaeidae

http://zoobank.org/57A2C8C3-2985-4255-B59F-DB5329A66490

#### Type material examined.

Holotype: ♂ [China] “Wang Tong, to light, 29/4/07” (ZFMK). Paratypes: 1 ♂ [China] “Huangniushi, Mt. Jiulianshan, Jiangxi, 16.VI.1975, leg. Zhang Youwei” (IZAS), 1 ♂ [China] “Luoyang, Lianxian County, Guangdong, 22.VI.1965, leg. Zhang Youwei” (IZAS).

#### Description.

Body length: 9.1 mm, length of elytra: 6.9 mm, width: 5.8 mm. Surface of labroclypeus and disc of frons glabrous. Smooth area anterior to eye twice as wide as long. Eyes moderately large; ratio of diameter/interocular width: 0.6. Antennal club 1.3 times as long as remaining antennomeres combined. Ratio of length of metepisternum/metacoxa: 1/1.7. Metafemur dull, anterior margin acute, without submarginal serrated line; anterior row of setae-bearing punctures absent; posterior margin straight, without blunt tooth. Metatibia short and wide, ratio width/length: 1/3.2; basal group of dorsal spines of metatibia at first third of metatibial length.

Aedeagus. Fig. 1I–K. Habitus: Fig. 1L.

Female unknown.

#### Variation.

Body length: 8.2–9.1 mm, length of elytra: 6.5–6.9 mm, width: 5.5–5.8 mm.

#### Diagnosis.

*Tetraserica wangtongensis* sp. n. is in the external shape and morphology of the male genitalia similar to *Tetraserica daqingshanica* and *Tetraserica sculptilis*. It differs by the lacking tooth at the posterior margin of metafemur and in the shape of the parameres: the right paramere is straight (in dorsal view) and not curved as in *Tetraserica daqingshanica* and *Tetraserica sculptilis* (Fig. 1J).

#### Etymology.

*Tetraserica wangtongensis* sp. n. is named after its type locality, Wang Tong.

### Tetraserica maoershanensis

Taxon classificationAnimaliaColeopteraScarabaeidae

Ahrens, Liu & Fabrizi sp. n.

http://zoobank.org/BBC43CD4-2FEE-431E-AD26-9B0D56CDC45B

#### Type material examined.

Holotype: ♂ “Guangxi, Maoershan, 2011-VI-4/ LW-1066” (ZFMK).

#### Description.

Body length: 8.3 mm, length of elytra: 6.8 mm, width: 3.1 mm.

Surface of labroclypeus and disc of frons glabrous. Smooth area anterior to eye twice as wide as long. Eyes moderately large; ratio of diameter/interocular width: 0.61. Antennal club 1.2 times as long as remaining antennomeres combined. Ratio of length of metepisternum/metacoxa: 1/1.5.

Metafemur dull, anterior margin acute, without submarginal serrated line; anterior row of setae-bearing punctures absent; posterior margin with a blunt tooth. Metatibia short and wide, ratio width/length: 1/2.84; basal group of dorsal spines of metatibia at first third of metatibial length.

**Figure 2.**
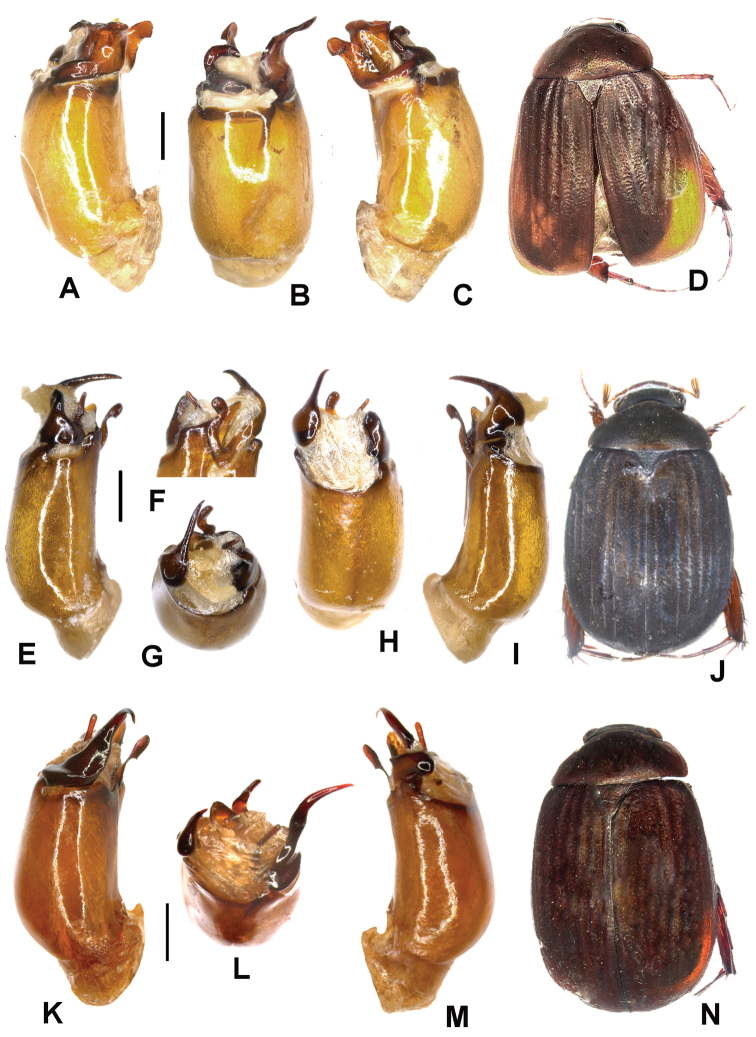
**A–D** *Tetraserica maoershanensis* sp. n. (holotype) **E–J** *Tetraserica fikaceki* sp. n. (holotype) **K–N** *Tetraserica changjiangensis* sp. n. (holotype). **A, E, K** aedeagus, left side lateral view **C, I, M** aedeagus, right side lateral view **B, H, L** parameres, dorsal view **F** parameres, distal view **G** parameres, ventral view **D, J, N** habitus (not to scale). Scale: 0.5 mm.

Aedeagus. Fig. 2A–C. Habitus: Fig. 2D.

Female unknown.

#### Diagnosis.

*Tetraserica maoershanensis* sp. n. is in the external shape and morphology of the male genitalia similar to *Tetraserica daqingshanica* and *Tetraserica sculptilis*. It differs by the shape of the parameres: the dorsal lobe of the right paramere is short and does not exceed the length of the ventral lobe (Fig. 2C).

#### Etymology.

The new species is named after its type locality, Maoershan.

### Tetraserica fikaceki sp. n.

Taxon classificationAnimaliaColeopteraScarabaeidae

http://zoobank.org/C23EC86C-5F90-4403-9617-826F307FB92C

#### Type material examined.

Holotype: ♂ “China, Hainan Isl., 4-6.v.2011 Limushan Mts. Frst administr. Centre (at light) 19°10'30"N, 109°44'33"E, 630m, M. Fikáček, V. Kubeček & L. Li leg.” (NMPC). Paratypes: 2 ♂♂, 1 ♀ [China] “Nankai Mingya, Baisha, Hainan, 25–26.V.2008, 450m, leg. Ba Yibin, Lang Juntong” (HBUM, ZFMK), 1 ♂ [China] “East of Mts. Bawangling, Changjiang, Hainan, 5-7.VI.2008, 750m, leg. Ba Yibin, Lang Juntong” (HBUM).

#### Description.

Body length: 9.7 mm, length of elytra: 7 mm, width: 6 mm.

Surface of labroclypeus and disc of frons glabrous. Smooth area anterior to eye twice as wide as long. Eyes moderately large; ratio of diameter/interocular width: 0.6. Antennal club 1.2 times as long as remaining antennomeres combined. Ratio of length of metepisternum/metacoxa: 1/1.8. Metafemur dull, anterior margin acute, without submarginal serrated line; anterior row of setae-bearing punctures absent; posterior margin straight, without blunt tooth. Metatibia short and wide, ratio width/length: 1/2.7; basal group of dorsal spines of metatibia at first third of metatibial length. Aedeagus. Fig. 2E–I. Habitus: Fig. 2J.

#### Diagnosis.

*Tetraserica fikaceki* sp. n. is in the external shape and morphology of the male genitalia similar to *Tetraserica wangtongensis*. It differs by the shape of the parameres: the dorsal lobe of the right paramere (in lateral view) is curved ventrally (while being straight in *Tetraserica wangtongensis*) (Fig. 2I), the left paramere is reduced in size (Fig. 2E).

#### Variation.

Body length: 8.0–9.7 mm, length of elytra: 6.0–7.0 mm, width: 4.8–6 mm. Head of the female paratype is missing.

#### Etymology.

The new species is named after one of the collectors of the type series, Martin Fikáček (Prague).

### Tetraserica changjiangensis sp. n.

Taxon classificationAnimaliaColeopteraScarabaeidae

http://zoobank.org/BCABBEB5-D1E5-4639-9042-64DF2FDD6E9B

#### Type material examined.

Holotype: ♂ “Bawangzhen, Changjiang, Hainan, 5–7.VI.2008, leg. Ba Yibin, Lang Juntong” (HBUM). Paratype: 1 ♂ “Mt. Jianfengling, Hainan, 10.VI.1965” (IZAS).

#### Description.

Body length: 8.4 mm, length of elytra: 6.5 mm, width: 5.4 mm.

Surface of labroclypeus and disc of frons glabrous. Smooth area anterior to eye twice as wide as long. Eyes moderately large; ratio of diameter/interocular width: 0.63. Antennal club 1.2 times as long as remaining antennomeres combined. Ratio of length of metepisternum/metacoxa: 1/1.67.

Metafemur dull, anterior margin acute, without submarginal serrated line; anterior row of setae-bearing punctures absent; posterior margin straight, without blunt tooth. Metatibia short and wide, ratio width/length: 1/2.74; basal group of dorsal spines of metatibia at first third of metatibial length.

Aedeagus. Fig. 2K–M. Habitus: Fig. 2N.

Female unknown.

#### Variation.

Body length: 7.7–8.4 mm, length of elytra: 6.1–6.5 mm, width: 5.0–5.4 mm.

#### Diagnosis.

*Tetraserica changjiangensis* sp. n. differs from the similar *Tetraserica fikaceki* sp. n. by the shape of the parameres: the dorsal lobe of the right paramere (in lateral view) is extremely short (Fig. 2M), the left paramere is distinctly longer and curved externally at the apex (Fig. 2K, L).

#### Etymology.

The new species is named after its type locality, Changjiang.

### Tetraserica sigulianshanica sp. n.

Taxon classificationAnimaliaColeopteraScarabaeidae

http://zoobank.org/745D0838-F4C7-42BA-8C16-A22ECAB1A0B8

#### Type material examined.

Holotype: ♂ “China: Sichuan; Wolong Reserve, Sigulian Shan, 31°09'N, 103°06'E v.2006, 1500–1800m leg. V. Siniaev” (ZFMK). Paratypes: 1 ♂ “Suifu (nr) Sz. China/ DC Graham coll. Aug 25-7, ‘29” (USNM), 1 ♂ “Szechuen China DC Graham/ bet Yachow and Mupin Jun.23-6 ‘29 2000-3000ft.” (USNM), 3 ♂♂ “Minzhuzhen, Lan'gao County, Shaanxi, 4.VII.2003, leg. Yuan Caixia, Liu Yushuang” (HBUM), 1 ♂ “Longju, Wanxian County, Sichuan, 18.VI.1995, 2500m, leg. Wang Shuyong” (IZAS), 1 ♂ “Foping, Shaanxi, 26.VI.1999, 890m, leg. Zhang Youwei” (IZAS), 1 ♂ “Chongqing, Jinfoshan, 2010-VI-13, 713m” (IZAS), 1 ♂ “Xiuqizhen, Chengkou, Chongqing, 13.VII.2003, leg. Yuan Caixia, Liu Yushuang” (HBUM), 1 ♂ “Ningshan, Shaanxi, VIII.1982, light trap” (NUYS), 1 ♂ “Zhongmiao, Bikou, Wenxian County, Gansu, 24.VI.1998, 700m, leg. Yuan Decheng” (IZAS), 1 ♂ “China, Sichuan 12.-14.VII.1995 Baoxing env., cca 50km NNW of Yaan 30°22'N, 102°50'E M. Trýzna et O. Šafranek lgt.” (CPPB).

#### Description.

Body length: 7.6 mm, length of elytra: 5.9 mm, width: 4.8 mm.

Surface of labroclypeus and disc of frons glabrous. Smooth area anterior to eye twice as wide as long. Eyes moderately large; ratio of diameter/interocular width: 0.6. Antennal club 1.2 times as long as remaining antennomeres combined. Ratio of length of metepisternum/metacoxa: 1/1.4.

Metafemur dull, anterior margin acute, without submarginal serrated line; anterior row of setae-bearing punctures absent; posterior margin straight, without blunt tooth. Metatibia short and wide, ratio width/length: 1/3; basal group of dorsal spines of metatibia at first third of metatibial length.

**Figure 3.**
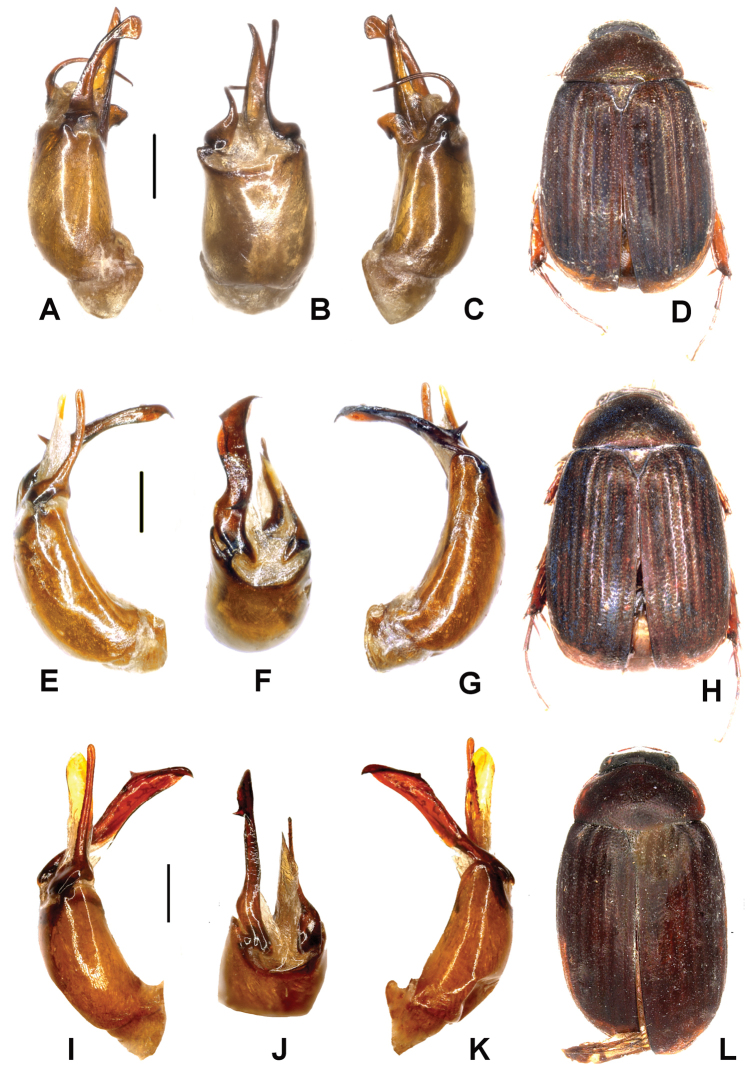
**A–D** *Tetraserica sigulianshanica* sp. n. (holotype) **E–H** *Tetraserica damaidiensis* sp. n. (holotype) **I–L** *Tetraserica shunbiensis* sp. n. (holotype). **A, E, I** aedeagus, left side lateral view **C, G, K** aedeagus, right side lateral view **B, F, J** parameres, dorsal view **D, H, L** habitus (not to scale). Scale: 0.5 mm.

Aedeagus. Fig. 3A–C. Habitus: Fig. 3D.

Female unknown.

#### Variation.

Body length: 6.6–7.6 mm, length of elytra: 5.1–5.9 mm, width: 3.9–4.8 mm.

#### Diagnosis.

*Tetraserica sigulianshanica* sp. n. differs from the similar *Tetraserica fikaceki* sp. n. by the shape of parameres: the dorsal (= basal) lobe of the right paramere (in lateral view) is long, filiform, and strongly curved (Fig. 3A), the left paramere is much longer than that of *Tetraserica fikaceki* sp. n. (Fig. 2K, L).

#### Etymology.

The new species is named after its type locality, Sigulian Shan.

### Tetraserica damaidiensis sp. n.

Taxon classificationAnimaliaColeopteraScarabaeidae

http://zoobank.org/48AB99AF-AF42-40A8-9775-9FE3054E1D22

#### Type material examined.

**Holotype:** ♂ “China: E-Yunnan; Damaidi 2500m, Guangnan near Vietnam VII-2003 leg. Li et al.” (ZFMK). Paratypes: **China.** 1 ♂ “China: E-Yunnan; Damaidi 2500m, Guangnan near Vietnam VII-2003 leg. Li et al.” (ZFMK), 1 ♂ “Jiangfu Famuchang, Jiangle, Fujian, 22.VI.1991, 470m, leg. Yang Longlong” (IZAS), 1 ♂ “Mt. Dawuling, Xinyi, 24.V.2002, leg. Jia Fenglong, No. En-048009” (SYUG), 2 ♂♂ “Luoxiang, Jinxiu, Guangxi, 15.V.1999, 400m, leg. Xiao Hui” (IZAS), 1 ♂ “Mt. Daweishan, Pingbian, Yunnan, 18.VI.1956, 1500m, light trap, leg. Huang Keren etc.” (IZAS), 1 ♂ “Mt. Yaoshan, 6.V.1938”(IZAS), 1 ♀ “Beidou, Napo, Guangxi, 9, 11–13.IV.1998, 550m, leg. Wu Min, Qiao Geixa” (IZAS), 1 ♂ “Nongxin, Napo, Guangxi, 12.IV.1998, 440m, leg. Qiao Gexia” (IZAS), 1 ♂ “Beidou, Napo, Guangxi, 9.IV.1998, 550m, leg. Qiao Gexia” (IZAS). **Vietnam.** 1 ♂ “N Vietnam (Tonkin) Ha Noi (city) 4.-5.V.1990 Vit. Kubáň leg. (ZFMK), 4 ♂♂, 10 ♀♀ “N-Vietnam Sa Pa env., Lao Cai Prov. 22°19'52"N, 103°50'35"E 1630–1680m 23.–27.V.1999 leg. Fabrizi, Jäger, Ahrens” (ZFMK), 1 ♂, 1 ♀ “N-Vietnam Bac Ha env., Lao Cai Prov. 22°32'05"N, 104°32'32"E 980–1000m 28.–30.V.1999 leg. Fabrizi, Jäger, Ahrens” (ZFMK), 2 ♀♀ “N.-Vietnam Fan Si Pan near Sapa, 1500–1950m 17.–30.VI.1999 A. Kallies leg.” (ZFMK), 1 ♂ “Vietnam N (Sa Pa) Lao Cai Prov., 250km from Hanoi bearing 31°, Sa Pa vill. env. Hoang Lien Son Nat. Res. 21.–23.6.1998 1250m leg. A. Napolov” (CNAR), 8 ♂♂ “Vietnam N (Sa Pa) Lao Cai Prov., 250km from Hanoi bearing 31°, Sa Pa vill. env. Hoang Lien Son Nat. Res. 27.V.–15.VI.1995 1250m leg. A. Napolov” (CNAR), 11 ♂♂ “Vietnam N (Sa Pa) Lao Cai Prov., 250km from Hanoi bearing 31°, Sa Pa vill. env. Hoang Lien Son Nat. Res. 27.5.–3.6.1998 1250m leg. A. Napolov” (CNAR), 1 ♂ “Vietnam N (Sa Pa) Lao Cai Prov., 250km from Hanoi bearing 31°, Sa Pa vill. env. Hoang Lien Son Nat. Res. 16.–20.VI.1998 1250m leg. A. Napolov” (CNAR).

#### Description.

Body length: 7.6 mm, length of elytra: 5.8 mm, width: 4.6 mm.

Surface of labroclypeus and disc of frons glabrous. Smooth area anterior to eye twice as wide as long. Eyes moderately large; ratio of diameter/interocular width: 0.67. Antennal club 1.3 times as long as remaining antennomeres combined. Ratio of length of metepisternum/metacoxa: 1/1.5. Metafemur dull, anterior margin acute, without submarginal serrated line; anterior row of setae-bearing punctures absent; posterior margin straight, without blunt tooth. Metatibia short and wide, ratio width/length: 1/3.21; basal group of dorsal spines of metatibia at first third of metatibial length.

Aedeagus. Fig. 3E–G. Habitus: Fig. 3H.

#### Variation.

Body length: 6.9–7.6 mm, length of elytra: 5.1–5.8 mm, width: 4.0–4.6 mm. Female has the antennal club composed of three lamellae, short, as long as the remaining antennomeres combined; eyes as large as those in male.

#### Diagnosis.

The new species differs from the other known *Tetraserica* species by having both parameres simple, not being divided in two lobes.

#### Etymology.

The new species is named after its type locality, Damaidi.

### Tetraserica shunbiensis sp. n.

Taxon classificationAnimaliaColeopteraScarabaeidae

http://zoobank.org/0E8B453F-D81F-484B-9897-7F615908507C

#### Type material examined.

Holotype: ♂ “Shunbi, Yangbi, Yunnan, 16.VIII.2009, leg. Shi Fuming” (HBUM).

#### Description.

Body length: 8.9 mm, length of elytra: 6.3 mm, width: 5 mm. Surface of labroclypeus and disc of frons glabrous. Smooth area anterior to eye twice as wide as long. Eyes moderately large; ratio of diameter/interocular width: 0.66. Antenna missing in holotype. Ratio of length of metepisternum/metacoxa: 1/1.5. Metafemur dull, anterior margin acute, without submarginal serrated line; anterior row of setae-bearing punctures absent; posterior margin straight, without blunt tooth. Metatibia short and wide, ratio width/length: 1/3.4; basal group of dorsal spines of metatibia at first third of metatibial length.

Aedeagus. Fig. 3I–K. Habitus: Fig. 3L.

Female unknown.

#### Diagnosis.

The new species differs from *Tetraserica damaidiensis* in the shape of the parameres: the left paramere is straight instead of being curved dorsally (Fig. 3I), the right paramere is slender in dorsal view, lacking the basal dorsal tooth (Fig. 3J) which is present in *Tetraserica damaidiensis*.

#### Etymology.

The new species is named after its type locality, Shunbi.

### Tetraserica longzhouensis sp. n.

Taxon classificationAnimaliaColeopteraScarabaeidae

http://zoobank.org/21E3E7F4-6784-45F3-8019-7DA174F598F0

#### Type material examined.

Holotype: ♂ [China] “Nonggang, Longzhou, Guangxi, 15.VI.2000, 330m, leg. Chen Jun” (IZAS).

#### Description.

Body length: 7.5 mm, length of elytra: 5.5 mm, width: 4.6 mm. Surface of labroclypeus and disc of frons glabrous. Smooth area anterior to eye twice as wide as long. Eyes small; ratio of diameter/interocular width: 0.48. Antennal club 1.1 times as long as remaining antennomeres combined. Ratio of length of metepisternum/metacoxa: 1/1.48. Metafemur dull, anterior margin acute, without submarginal serrated line; anterior row of setae-bearing punctures absent; posterior margin straight, without blunt tooth. Metatibia short and wide, ratio width/length: 1/3.3; basal group of dorsal spines of metatibia at first third of metatibial length.

**Figure 4.**
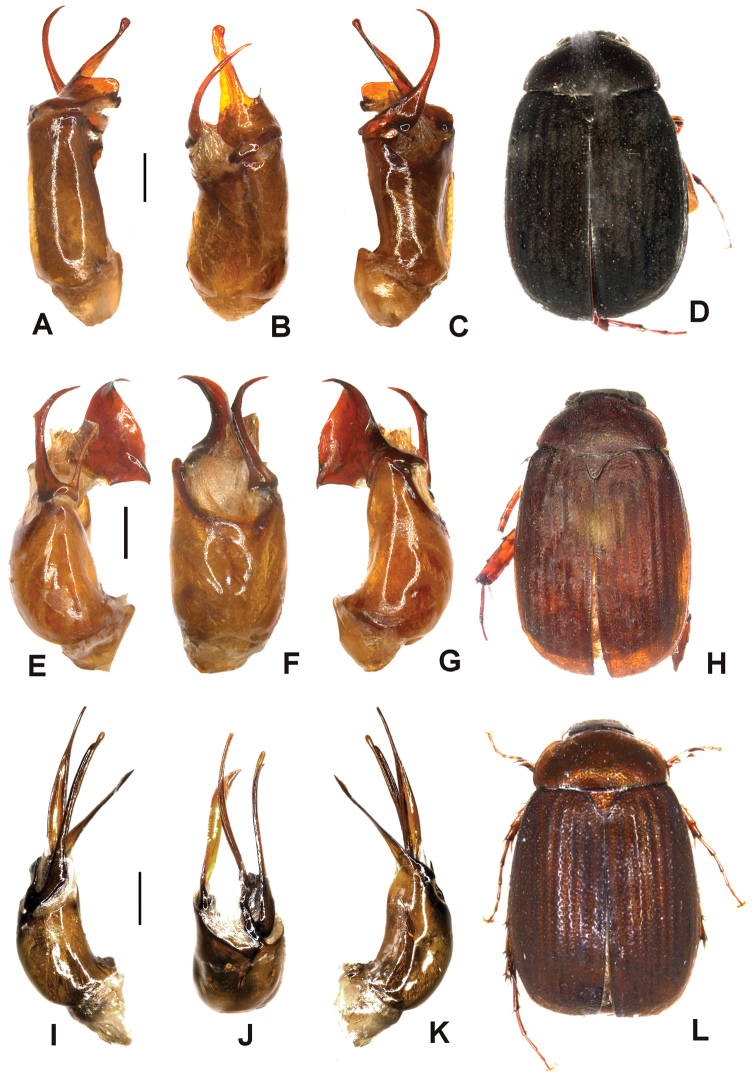
**A–D** *Tetraserica longzhouensis* sp. n. (holotype) **E–H** *Tetraserica yaoquensis* sp. n. (holotype) **I–L** *Tetraserica longipenis* sp. n. (holotype). **A, E, I** aedeagus, left side lateral view **C, G, K** aedeagus, right side lateral view **B, F, J** parameres, dorsal view **D, H, L** habitus (not to scale). Scale: 0.5 mm.

Aedeagus. Fig. 4A–C. Habitus: Fig. 4D.

Female unknown.

#### Diagnosis.

*Tetraserica longzhouensis* sp. n. differs from all other species with straight or slightly convex posterior margin of metafemur by the small eyes, short ventral process of phallobasis being at maximum subequeal to half of the length of the phallobasis, strongly asymmetric phallobasis (dorsal view), right paramere being simple, and left paramere having the ventral lobe shorter than the dorsal one.

#### Etymology.

The new species is named after its type locality, Longzhou.

### Tetraserica yaoquensis sp. n.

Taxon classificationAnimaliaColeopteraScarabaeidae

http://zoobank.org/90F90FBE-55EC-4183-B7F3-41659BC1E687

#### Type material examined.

Holotype: ♂ [China] “Yao District, Mengla, Yunnan, 11.V.1991, leg. Liu Guangchun, Cai Wanzhi” (NUYS).

#### Description.

Body length: 8.3 mm, length of elytra: 6.5 mm, width: 5.1 mm. Surface of labroclypeus and disc of frons glabrous. Smooth area anterior to eye twice as wide as long. Eyes moderately large; ratio of diameter/interocular width: 0.62. Antennal club 1.6 times as long as remaining antennomeres combined. Ratio of length of metepisternum/metacoxa: 1/1.5. Metafemur dull, anterior margin acute, without submarginal serrated line; anterior row of setae-bearing punctures absent; posterior margin straight, without blunt tooth. Metatibia short and wide, ratio width/length: 1/3.2; basal group of dorsal spines of metatibia at first third of metatibial length.

Aedeagus. Fig. 4E–G. Habitus: Fig. 4H.

Female unknown.

#### Diagnosis.

*Tetraserica yaoquensis* sp. n. differs from all other species with straight posterior margin of metafemur by the short ventral process of the phallobasis being subequal to half of the length of phallobasis, right paramere being simple and basiventrally strongly widened towards apex, and left paramere having two lobes.

#### Etymology.

The new species is named after its type locality, Yaoqu.

### Tetraserica longipenis sp. n.

Taxon classificationAnimaliaColeopteraScarabaeidae

http://zoobank.org/DF2660FC-8E23-4C34-9E1E-986936B883C3

#### Type material examined.

Holotype: ♂ “China: E-Yunnan; Damaidi 2500m, Guangnan near Vietnam VII-2003 leg. Li et al.” (ZFMK). Paratypes: 1 ♂ “China, SE-Yunnan Xichou-E env. 1400–1700m, 13.–18.5.95/ 23°11–16'/ 104°41–49' L.+R. Businský lgt.” (CPPB), 1 ♂ [China] “Yunnan 2000–2500m 25.42N 100.08E Cangshan mts. E slope 21.VI.92 David Král leg.” (NMPC), 1 ♂ [China] “Mts. Laoshan, Weihuo, Tianlin, Guangxi, 4.VI.2002, 1400m, leg. Jiang Guofang” (IZAS), 1 ♂ [China] “Luodian, Guizhou, 29.V.1981, 500m, leg. Li Fasheng” (CAU), 2 ♂♂ [China] “Xinyicun, Xichang, Sichuan, 18.V.1974, leg. Han Yinheng” (IZAS), 3 ♂♂ [China] “Meng'e, Xishuangbanna, Yunnan, 19.V.1958, leg. Hong Chunpei” (IZAS, ZFMK).

#### Description.

Body length: 7 mm, length of elytra: 5.3 mm, width: 4.6 mm. Surface of labroclypeus and disc of frons glabrous. Smooth area anterior to eye twice as wide as long. Eyes moderately large; ratio of diameter/interocular width: 0.7. Antennal club 1.2 times as long as remaining antennomeres combined. Ratio of length of metepisternum/metacoxa: 1/1.4. Metafemur dull, anterior margin acute, without submarginal serrated line; anterior row of setae-bearing punctures absent; posterior margin straight, without blunt tooth. Metatibia short and wide, ratio width/length: 1/3.3; basal group of dorsal spines of metatibia at first third of metatibial length.

Aedeagus. Fig. 4E–G. Habitus: Fig. 4H.

Female unknown.

#### Variation.

Body length: 7.0–7.8 mm, length of elytra: 5.3–5.6 mm, width: 4.4–4.6 mm.

#### Diagnosis.

*Tetraserica longipenis* sp. n. differs from *Tetraserica yaoquensis* by the smaller body, shorter antennal club, and the right paramere being simple, long and narrow.

#### Etymology.

The species name is derived from the combined Latin words, *longus* – long and *penis* – male copulation organ, with reference to the long parameres of the species.

### Tetraserica jinghongensis sp. n.

Taxon classificationAnimaliaColeopteraScarabaeidae

http://zoobank.org/BFE3EECD-6FE2-4C1D-9D70-F80CEF0D1F31

#### Type material examined.

Holotype: ♂ “China, S-Yunnan Prov. Xishuangbanna 23km NW Jinghong Na Ban village 680m 22°10.04'N, 100°39.52'E, 20.V.2008, leg. A. Weigel, LF” (NME). Paratypes. 1 ♂ [China] “Xiaomengyang, Yunnan, 7.V.1957, 850m, leg. Pu Fuji” (IZAS), 1 ♂ [China] “Yunnan, Nabanhe Nature Reserve, Guomenshan, 2009-VI-16/ LW-1230” (ZFMK).

#### Description.

Body length: 7.6 mm, length of elytra: 5.8 mm, width: 5 mm. Surface of labroclypeus and disc of frons glabrous. Smooth area anterior to eye twice as wide as long. Eyes moderately large; ratio of diameter/interocular width: 0.63. Antenna missing in holotype. Ratio of length of metepisternum/metacoxa: 1/1.69. Metafemur dull, anterior margin acute, without submarginal serrated line; anterior row of setae-bearing punctures absent; posterior margin straight, without blunt tooth. Metatibia short and wide, ratio width/length: 1/3.36; basal group of dorsal spines of metatibia at first third of metatibial length.

**Figure 5.**
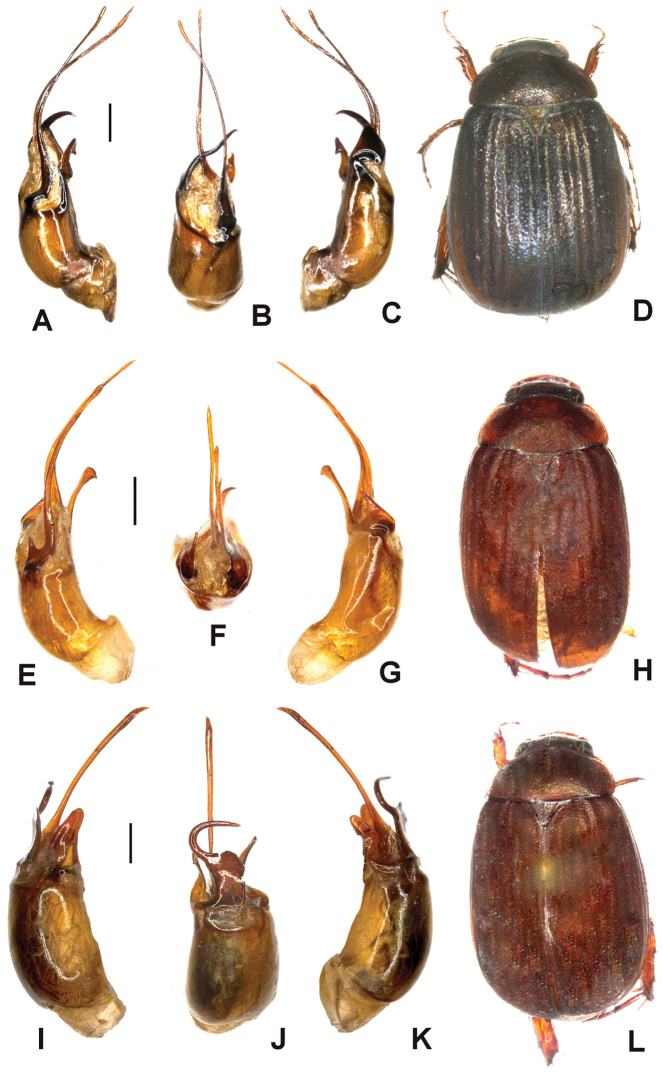
**A–D** *Tetraserica jinghongensis* sp. n. (holotype) **E–H** *Tetraserica menglongensis* sp. n. (holotype) **I–L** *Tetraserica tianchiensis* sp. n. (holotype). **A, E, I** aedeagus, left side lateral view **C, G, K** aedeagus, right side lateral view **B, F, J** parameres, dorsal view **D, H, L** habitus (not to scale). Scale: 0.5 mm.

Aedeagus. Fig. 5A–C. Habitus: Fig. 5D.

Female unknown.

#### Variation.

Body length: 6.8–7.6 mm, length of elytra: 5.2–5.8 mm, width: 4.1–5.0 mm.

#### Diagnosis.

*Tetraserica jinghongensis* sp. n. differs from all other species with long ventral phallobasal process by the simple left paramere being not separated into lobes, right paramere having no brush of spines and being composed of two lobes: its dorsal lobe is narrow, evenly curved and sharply pointed.

#### Etymology.

The species name is named after its type locality, Jinghong Na Ban village.

### Tetraserica menglongensis sp. n.

Taxon classificationAnimaliaColeopteraScarabaeidae

http://zoobank.org/D5152BD3-A0F2-4A16-9A8C-DCBC8322BC0F

#### Type material examined.

Holotype: ♂ [China] “Menglong, Yunnan, 22.IV.1982, light trap, leg. Jiang Shengqiao” (IZAS).

#### Description.

Body length: 7.6 mm, length of elytra: 5.8 mm, width: 4.3 mm. Surface of labroclypeus and disc of frons glabrous. Smooth area anterior to eye twice as wide as long. Eyes moderately large; ratio of diameter/interocular width: 0.59. Antennal club 1.2 times as long as remaining antennomeres combined. Ratio of length of metepisternum/metacoxa: 1/1.64. Metafemur dull, anterior margin acute, without submarginal serrated line; anterior row of setae-bearing punctures absent; posterior margin straight, without blunt tooth. Metatibia short and wide, ratio width/length: 1/3.23; basal group of dorsal spines of metatibia at first third of metatibial length.

Aedeagus. Fig. 5E–G. Habitus: Fig. 5H.

Female unknown.

#### Diagnosis.

*Tetraserica menglongensis* sp. n. differs from *Tetraserica jinghongensis* by the slightly lighter colour, bilobate left paramere, as well as the right paramere having the dorsal lobe shorter than the ventral one (Fig. 5E).

#### Etymology.

The new species is named after its type locality, Menglong.

### Tetraserica tianchiensis sp. n.

Taxon classificationAnimaliaColeopteraScarabaeidae

http://zoobank.org/8BB6DBDE-F5BC-4808-9BE2-3459B99E377D

#### Type material examined.

Holotype: ♂ “Tianchi, Jianfeng, Hainan, 13.IV.1980, 900m, leg. Wang Shuyong” (IZAS). Paratype: 1 ♂ “Tianchi, Jianfeng, Hainan, 18.IV.1980, 750m, leg. Wang Shuyong” (ZFMK).

#### Description.

Body length: 9.1 mm, length of elytra: 6.9 mm, width: 5.7 mm. Surface of labroclypeus and disc of frons glabrous. Smooth area anterior to eye twice as wide as long. Eyes moderately large; ratio of diameter/interocular width: 0.63. Antennal club 1.3 times as long as remaining antennomeres combined. Ratio of length of metepisternum/metacoxa: 1/1.56. Metafemur dull, anterior margin acute, without submarginal serrated line; anterior row of setae-bearing punctures absent; posterior margin straight, without blunt tooth. Metatibia short and wide, ratio width/length: 1/3.24; basal group of dorsal spines of metatibia at first third of metatibial length.

Aedeagus. Fig. 5I–K. Habitus: Fig. 5L.

Female unknown.

#### Variation.

Body length: 9.1–10.5 mm, length of elytra: 6.9–8.4 mm, width: 5.7–6.1 mm.

#### Diagnosis.

*Tetraserica tianchiensis* sp. n. differs from *Tetraserica jinghongensis* by dorsal lobe of the right paramere being wide, with a sickle-shaped, large apical hook (Fig. 5J).

#### Etymology.

The new species is named after its type locality, Tianchi.

### Tetraserica liangheensis sp. n.

Taxon classificationAnimaliaColeopteraScarabaeidae

http://zoobank.org/D3CCF90A-4B08-4141-A735-D2D311ABE1D1

#### Type material examined.

Holotype: ♂ [China] “Yunnan, Lianghe, 2011-V-4, N: 24.789, E: 98.264, 1130m/ LW-1316” (ZFMK).

#### Description.

Body length: 9.7 mm, length of elytra: 7.3 mm, width: 5.5 mm. Surface of labroclypeus and disc of frons glabrous. Smooth area anterior to eye twice as wide as long. Eyes moderately large; ratio of diameter/interocular width: 0.59. Antennal club 1.1 times as long as remaining antennomeres combined. Ratio of length of metepisternum/metacoxa: 1/1.56. Metafemur dull, anterior margin acute, without submarginal serrated line; anterior row of setae-bearing punctures absent; posterior margin with a large sharp hook. Metatibia short and wide, ratio width/length: 1/3.1; basal group of dorsal spines of metatibia at first third of metatibial length.

**Figure 6.**
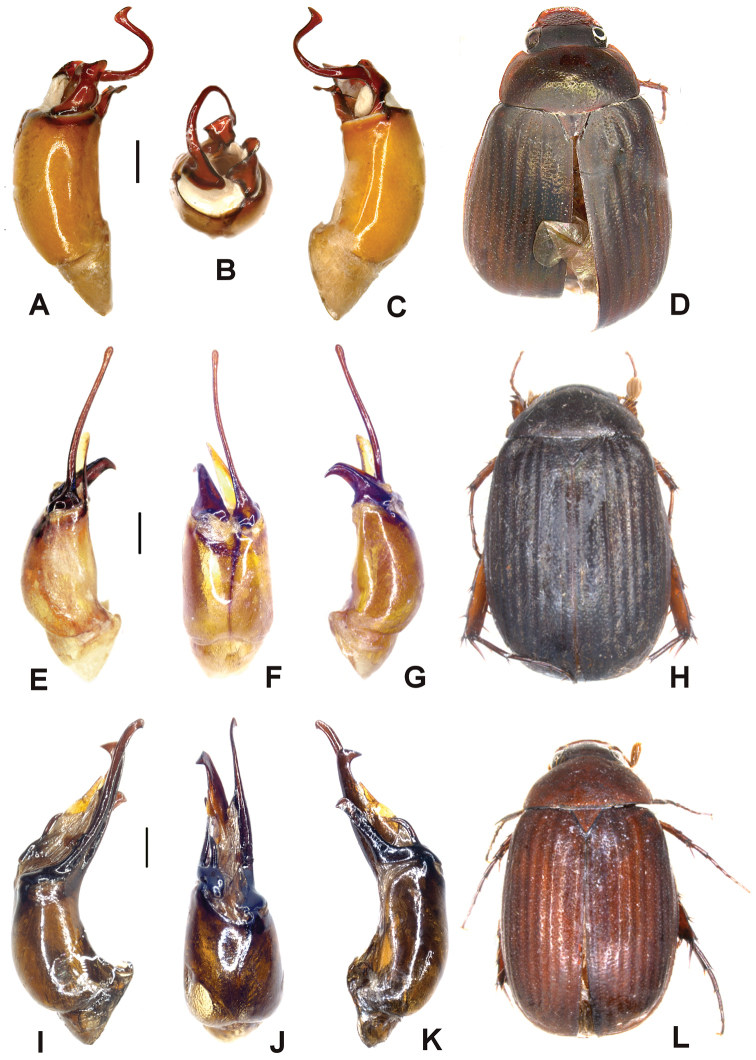
**A–D** *Tetraserica liangheensis* sp. n. (holotype) **E–H** *Tetraserica graciliforceps* sp. n. (holotype) **I–L** *Tetraserica pingjiangensis* sp. n. (holotype). **A, E, I** aedeagus, left side lateral view **C, G, K** aedeagus, right side lateral view **B, F, J** parameres, dorsal view **D, H, L** habitus (not to scale). Scale: 0.5 mm.

Aedeagus. Fig. 6A–C. Habitus: Fig. 6D.

Female unknown.

#### Diagnosis.

*Tetraserica liangheensis* sp. n. differs from all other known *Tetraserica* species by the large and sharp hook at the posterior margin of metafemur.

#### Etymology.

The new species is named after its type locality, Lianghe.

### Tetraserica graciliforceps sp. n.

Taxon classificationAnimaliaColeopteraScarabaeidae

http://zoobank.org/63505155-B061-49FC-B407-E0BD37477162

#### Type material examined.

Holotype: ♂ “China, W Yunnan prov., mts. 60km E Tengchong, 2200m, 19.–22.v.2006, S. Murzin & I. Shokhin” (CPPB). Paratype: 1 ♂ “Yunnan 2200-2500m 24.57N 98.45E 8-16/5. Gaoligong mts. Vit Kubáň leg. 1995” (ZFMK).

#### Description.

Body length: 8.9 mm, length of elytra: 7.2 mm, width: 5.7 mm. Surface of labroclypeus and disc of frons glabrous. Smooth area anterior to eye twice as wide as long. Eyes small; ratio of diameter/interocular width: 0.5. Antennal club 1.2 times as long as remaining antennomeres combined. Ratio of length of metepisternum/metacoxa: 1/1.6. Metafemur dull, anterior margin acute, without submarginal serrated line; anterior row of setae-bearing punctures absent; posterior margin with a large sharp hook. Metatibia short and wide, ratio width/length: 1/3.2; basal group of dorsal spines of metatibia at first third of metatibial length.

Aedeagus. Fig. 6E–G. Habitus: Fig. 6H.

Female unknown.

#### Variation.

Body length: 8.9–9.8 mm, length of elytra: 7.1–7.2 mm, width: 5.5–5.7 mm.

#### Diagnosis.

*Tetraserica graciliforceps* sp. n. differs from *Tetraserica longzhouensis* by the following features: phallobasis in dorsal view is only slightly asymmetric; left and right parameres are simple, without two lobes; posterior angles of the pronotum are strongly rounded.

#### Etymology.

The species name is derived from the combined Latin words, *gracilis* – fine, and *forceps* – forceps, with reference to the fine and simple parameres of the species.

### Tetraserica pingjiangensis sp. n.

Taxon classificationAnimaliaColeopteraScarabaeidae

http://zoobank.org/02009348-3716-4747-B9AD-9B82890893C9

#### Type material examined.

Holotype: ♂ “China: Hunan; Mupu Mt. 1600m, Pingjiang VIII-2003, leg. Li et al.” (ZFMK). Paratypes: **China.** 1 ♀ “China: Hunan; Mupu Mt. 1600m, Pingjiang VIII-2003, leg. Li et al.” (ZFMK), 2 ♂♂, 1 ♀ “China: Hubei; Dahongshan 1700m Shuizhou VI-2003 leg. Ying et al.” (ZFMK), 1 ♂ “China: S-Yunnan Prov. Xishuangbanna 20km NW Jinghong Man Dian (NNNR) 720m 22°07.80'N, 100°40.05'E, 26.v.2008, light trap, leg. A. Weigel” (NME), 1 ♂ “Jingdong, Yunnan, 30.V.1956, 1170m, leg. Krischanovskna” (IZAS), 2 ♂♂ [China] “Jingdong, Yunnan, 22.V, 1.VI.1956, 1170m, leg. Zagulaev, Polov” (IZAS), 2 ♂♂ [China] “Mt. Junzishan, Shizong, Yunnan, 14,16.VII.2006, leg. Mao Benyong etc.” (HBUM), 1 ♂ “Jingdong, Yunnan, 29.IV.1957, 1200m, leg. Zagulaev” (IZAS), 1 ♂ [China] “Yunnan, Honghe, Hekou, Binlangzhai Shuiku, 2011-V-14/ LW-1076” (IZAS), 4 ♂♂ [China] “Jingdong, Yunnan, 26,30.V.1956, 1170m, leg. Krischanovskna, light trap” (IZAS), 3 ♂♂ [China] “Jingdong, Yunnan, 1,3.VII.1956, 1170m, leg. Krischanovskna, light trap” (IZAS), 8 ♂♂ [China] “Jingdong, Yunnan, 2,5,8,12,20,21,28.VI.1956, 1170m, leg. Krischanovskna, light trap” (IZAS), 1 ♂ [China] “Jingdong, Yunnan, 17.V.1957, 1200m, leg. Montschadskij” (IZAS), 1 ♂ [China] “Jingdong, Yunnan, 8.IV.1957, 1200m, leg. Montschadskij” (IZAS), 7 ♂♂, 1 ♀ [China] “Jingdong, Yunnan, 10.V.1957, 1200m, leg. Montschadskij” (IZAS), 1 ♂ [China] “22 km Northeast of Jingdong, 12.V.1957,leg. Montschadskij” (IZAS), 4 ♂♂ [China] “Jingdong, Yunnan, 23,31.V.1956, 1170–1300m, leg. Ivanov, light trap” (IZAS), 2 ♂♂ [China] “Jingdong, Yunnan, 3.VI.1956, 1170m, leg. Zagulaev” (IZAS), 4 ♂♂ [China] “Jingdong, Yunnan, 30.VI.1956, 1170m, leg. Zagulaev” (IZAS), 1 ♂ [China] “Dongjiafen, Jingdong, Yunnan, 27.VI.1956, 1250m, leg. A. Shnitnikov” (IZAS), 1 ♂ [China] “Fulong, Fangcheng, Guangxi, 23.V.1999, 200m, leg. Ke Xin” (IZAS), 1♂ [China] “Fulong, Fangcheng, Guangxi, 25.V.1999, 200m, leg. Zhang Xuezhong” (IZAS), 1 ♂ [China] “Hongqi Forestry Farm, Shangsi, Guangxi, 28.V.1999, 300m, leg. Zhang Xuezhong” (IZAS), 3 ♂♂ [China] “Banbaxiang, Fangcheng, Guangxi, 16.V.2000, 550m, leg. Li Wenzhu” (IZAS), 2 ♂♂ [China] “Huanian (Eshan, Yunnan), 15.VI.(19)83, No.312,319” (IZAS), 3 ♂♂ [China] “Mengla, Jinping, Yunnan, 17,20.IV.1956, 370m, leg. Huang Keren et.al.” (IZAS), 2 ♂♂ [China] “Menglun, Yunnan, 22.IV.1982, leg. Jiang, light trap” (IZAS), 1 ♂ [China] “Menglun, Yiwubanna, 8.VII.1964, 650m, leg. Zhang Baolin” (IZAS). **Myanmar.** 1 ♂ “Burma (Myanmar) E Shan state Kengtung (Kyaingtong) J. Rejsek 14.–15.6.1997” (ZFMK). **Vietnam.** 1 ♂ “N Vietnam (Tonkin) Ha Noi (city) 4.–5.V.1990 Vit. Kubáň leg. (ZFMK), 1 ♂ “Vietnam, Cuc Phong, 100km SE of Hanoi; V-1993; leg. Michio Hori” (ZFMK), 1 ♂ “Vietnam N. Tonkin Cuc-Phong Nat. Park 2.–12.V.1991 E. Jendek leg.” (ZFMK), 45 ♂♂, 44 ♀♀ “N-Vietnam Bac Ha env., Lao Cai Prov., 22°32‘05‘'N, 104°17‘32‘‘E 980-1000m 28.–30.V.1999 leg. Fabrizi, Jager, Ahrens” (ZFMK), 1 ♂ “N. Vietnam: Sontay, 400m, 20.V.1963 leg. Le Van Dyk” (Coll. Kabakov), 1 ♂ “N. Vietnam: Sonla Songma, 500m, 3.V.1990 leg. Schorkov” (Coll. Kabakov), 1 ♂ “N. Vietnam: 40km NE Thainguyen, 300m, 8.V.1963, leg. O. Kabakov” (Coll. Kabakov), 1 ♂ “S. Vietnam (Cat Tien) 120 km NNE Ho Chi Minh, Cat Tien Nat. Park 30.5.–15.6.1995 leg. A. Napolov” (CNAR), 17 ♂♂ “Vietnam N (Sa Pa) Lao Cai Prov., 250km from Hanoi bearing 31°, Sa Pa vill. env. Hoang Lien Son Nat. Res. 27.5.–3.6.1998 1250m leg. A. Napolov” (CNAR, ZFMK), 2 ♂♂ “Vietnam N (Sa Pa) Lao Cai Prov., 250km from Hanoi bearing 31°, Sa Pa vill. env. Hoang Lien Son Nat. Res. 27.V.-3.VI.1998 1250m leg. A. Napolov” (CNAR, ZFMK), 1 ♂ “Vietnam N (Sa Pa) Lao Cai Prov., 250km from Hanoi bearing 31°, Sa Pa vill. env. Hoang Lien Son Nat. Res. 16.–20.VI.1998 1250m leg. A. Napolov” (CNAR), 3 ♂♂ “Vietnam N (Sa Pa) Lao Cai Prov., 250km from Hanoi bearing 31°, Sa Pa vill. env. Hoang Lien Son Nat. Res. 25.VI.–5.VII.1998 1250m leg. A. Napolov” (CNAR), 3 ♂♂ “Vietnam N (Sa Pa) Lao Cai Prov., 250km from Hanoi bearing 31°, Sa Pa vill. env. Hoang Lien Son Nat. Res. 21.–23.6.1998 1250m leg. A. Napolov” (CNAR), 3 ♂♂ “Vietnam-N (Na Hang) 160km from Ha Noi, NE env. of Na Hang, 26.5.–6.6.1996 150–200m leg. A. Napolov & I. Roma” (CNAR), 1 ♂ “X-DA3434 - Vietnam, N. Vietnam: Hanoi Prov., Ba Vi National Park (at light) 21–24.vi.2012 L. Bartolozzi, S. Bambi, F. Fabiano, E. Orbach” (MZUF). **Thailand.** 2 ♂♂, 3 ♀♀ “Thailand, Nam Nao, Phetchabua; 16°5'N,101°40'E; 19.V.1999; leg. K. Masumoto” (ZFMK), 1 ♂, 1 ♀ “Phu Rua NP (900m alt.), Loei P., NE Thai. 26–30.IV.2006 Takakuwa, M. leg.” (ZFMK), 3 ♂♂, 1 ♀ “N. Thailand, Chiang Mai, Erawan Resort; 22.IV.1992; leg. Kazuo Kawano” (ZFMK), 1 ♂, 2 ♀♀ “N. Thailand, Nan, Wiang Sa; 14.V.1993; leg. S. Ohmomo” (ZFMK), 1 ♂ “839493 Tetraserica ThaiSpMU09_1 Thailand S. Murzin 28./31.5.2009 Chiang Dao Hill resort (100km N of Chiang Mai) 600m/ 839493” (ZFMK), 1 ♂, 2 ♀♀ “Thailand bor. Chiang Dao env., 21.5.–4.6.1995 lgt. M. Snizek” (ZFMK), 2 ♂♂ “NW Thailand 24.-27.4.1991 Chom Thong leg. Pacholátko/ TS98” (CPPB), 1 ♂ “Thai 11–15.V.1998 Nan-Pha Khab Pacholátko & Dembický leg.” (CPPB), 1 ♂ “NW Thailand, 1991 Chow Thong, 24.–27.4. 18.26N, 98.41E L. Dembicky leg.” (NHMW), 1 ♂ “Thailand Chiangmai Prov.; Fang (Agr Exp. Station), 600 m 14.VI.1965” (BPBM), 2 ♂♂, 1 ♀ “N. Thailand: Angkhai village, Samoeng Dist. Chiang Mai Prov., 9–11.v.1999 K. Masumoto leg,” (ZFMK), 1 ♂ “N. Thailand: Chiang Mai Pref., Ban Angkhai, Samoeng Dist., 750 m, 15–20.V.1998 K. Matsumoto leg.” (ZFMK). **Laos.** 1 ♂ “Laos, 21°09'N, 101°19'E Louangnamtha pr. Namtha → Muang Sing, 5-31.v.1997, 900-1200m Vit Kubáň leg./ LS11” (CPPB), 1 ♂ “Laos centr., 27.IV.–1.V.1997, 70km NE Vientiane Ban Phabat env., 150m, N18°16.1', E103°10.9' E. Jendek & Šauša leg.” (CPPB), 1 ♂ “Laos, Bolikhamxai pr. 18°16'N, 103°11'E 70km NEE Vientiane, 27-30.iv.1997, 150m, Vit Kubáň leg.” (CPPB), 2 ♂♂ “N. Laos Louang Namtha 1.5.1996 I. Pjushtch lg” (ZFMK), 35 ♂♂, 19 ♀♀ “Laos P.D.R. Xieng Khowang 14–20. May 1994 K. Miura leg.” (ZFMK), 2 ♂♂ “NE-Laos: Hua Phan prov.; Ban Saleui, Phou Pan (Mt.) 20°12'N, 104°01'E 11.iv.–15.v.2012, 1300–1900 m leg. C. Holzschuh → ZFMK Ankauf 2012/13” (ZFMK), 5 ♂♂ “Laos, La Oudomxay 16.6.2005, ex. coll. Sabatinelli” (ZFMK), 4 ♂♂ “Laos-N, Louang Namtha circ. 04.05.1996, I. Pjushtch lg” (ZFMK).

#### Description.

Body length: 10.0 mm, length of elytra: 7.5 mm, width: 6.0 mm. Surface of labroclypeus and disc of frons glabrous. Smooth area anterior to eye twice as wide as long. Eyes moderately large; ratio of diameter/interocular width: 0.6. Antennal club 1.2 times as long as remaining antennomeres combined. Ratio of length of metepisternum/metacoxa: 1/1.6. Metafemur dull, anterior margin acute, without submarginal serrated line; anterior row of setae-bearing punctures absent; posterior margin with a blunt tooth. Metatibia short and wide, ratio width/length: 1/2.9; basal group of dorsal spines of metatibia at first third of metatibial length.

Aedeagus. Fig. 6I–K. Habitus: Fig. 6L.

#### Diagnosis.

*Tetraserica pingjiangensis* sp. n. differs from all other *Tetraserica* species with a blunt tooth at the posterior margin of metafemur by the long and narrow left paramere.

#### Etymology.

The new species is named after its type locality, Pingjiang.

#### Variation.

Body length: 8.8–10.0 mm, length of elytra: 6.9–7.5 mm, width: 5.4–6.0 mm. Female has the antennal club composed of three lamellae, short, as long as remaining antennomeres combined; eyes are as large as those in male.

### Tetraserica wandingensis sp. n.

Taxon classificationAnimaliaColeopteraScarabaeidae

http://zoobank.org/D422148E-C6DA-4584-8F5E-07FD832F4CE3

#### Type material examined.

Holotype: ♂ [China] “Yunnan, Wanding, 2011-IV-29, N: 24.086, E: 98.072, 900m/ LW-1247” (ZFMK). Paratype: 1 ♂ [China] “Yunnan, Wanding, 2011-IV-29, N: 24.086, E: 98.072, 900m/ LW-1247bis” (IZAS).

#### Description.

Body length: 9.3 mm, length of elytra: 6.9 mm, width: 5.4 mm. Surface of labroclypeus and disc of frons glabrous. Smooth area anterior to eye twice as wide as long. Eyes large; ratio of diameter/interocular width: 0.72. Antennal club 1.5 times as long as remaining antennomeres combined. Ratio of length of metepisternum/metacoxa: 1/1.56. Metafemur dull, anterior margin acute, without submarginal serrated line; anterior row of setae-bearing punctures absent; posterior margin with a large sharp hook. Metatibia short and wide, ratio width/length: 1/3.13; basal group of dorsal spines of metatibia at first third of metatibial length.

**Figure 7.**
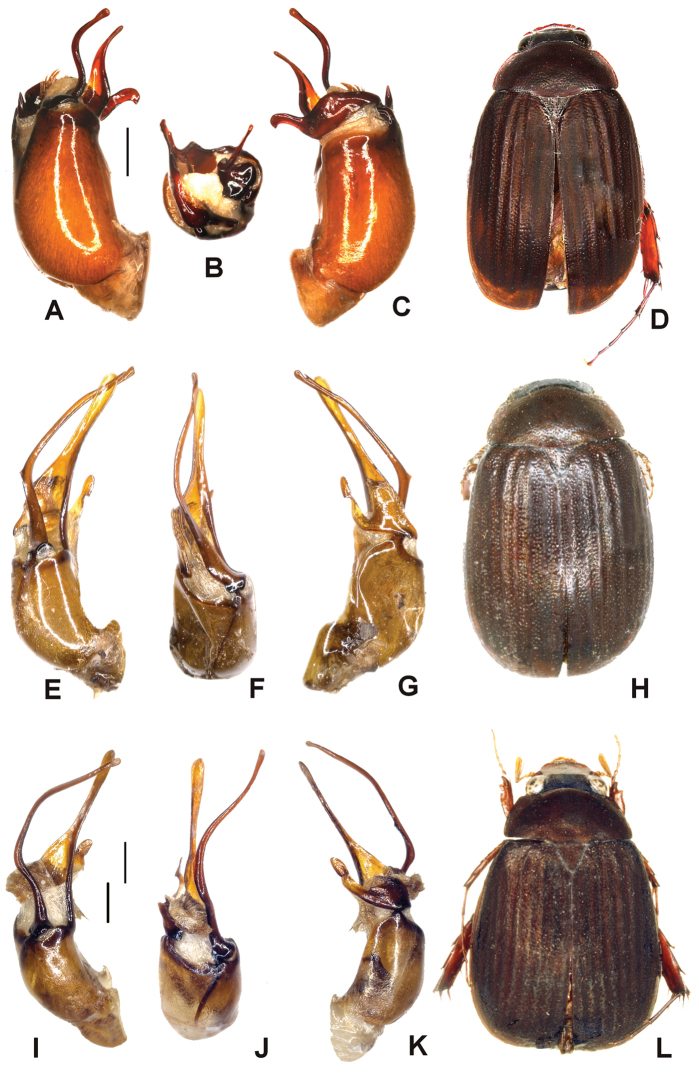
**A–D** *Tetraserica wandingensis* sp. n. (holotype) **E–H** *Tetraserica tonkinensis* (Moser) comb. n. (lectotype) **I–L** *Tetraserica xichouensis* sp. n. (holotype). **A, E, I** aedeagus, left side lateral view **C, G, K** aedeagus, right side lateral view; **B, F, J** parameres, dorsal view **D, H, L** habitus (not to scale). Scale: 0.5 mm.

Aedeagus. Fig. 7A–C. Habitus: Fig. 7D.

Female unknown.

#### Variation.

Body length: 8.8–9.3 mm, length of elytra: 6.9–7.1 mm, width: 5.4–5.6 mm.

#### Diagnosis.

*Tetraserica wandingensis* sp. n. differs from *Tetraserica liangheensis* by the larger eyes (ratio diameter/interocular distance: 0.72 vs. 0.59) and dorsal lobe of the right paramere being very small and bent basally.

#### Etymology.

The new species is named after the type locality, Wanding.

### Tetraserica tonkinensis

Taxon classificationAnimaliaColeopteraScarabaeidae

(Moser, 1908) comb. n.

Neoserica tonkinensis Moser, 1908: 328 (type locality: Tonkin, Mt.Mauson).

#### Type material examined.

Lectotype (here designated): ♂ [Vietnam] “Tonkin Montes Mauson April, Mai 2-3000' H. Fruhstorfer/ tonkinensis Mos.” (ZMHB). Paralectotypes: 1 ♀ [Vietnam] “Tonkin Montes Mauson April, Mai 2-3000' H. Fruhstorfer/ tonkinensis Mos.” (ZMHB), 5 ♀♀ [Vietnam] “Tonkin Montes Mauson April, Mai 2-3000' H. Fruhstorfer” (ZMHB).

#### Additional material examined.

1 ♂ [China] “Luoxiang, Jinxiu, Guangxi, 15.V.1999, 400m, leg. Li Wenzhu” (IZAS).

#### Redescription.

Body length: 9.0 mm, length of elytra: 7.0 mm, width: 5.9 mm. Surface of labroclypeus and disc of frons glabrous. Smooth area anterior to eye twice as wide as long. Eyes large; ratio of diameter/interocular width: 0.66. Antennal club 1.2 times as long as remaining antennomeres combined. Ratio of length of metepisternum/metacoxa: 1/1.45. Metafemur dull, anterior margin acute, without submarginal serrated line; anterior row of setae-bearing punctures absent; posterior margin with a large sharp hook. Metatibia short and wide, ratio width/length: 1/3.25; basal group of dorsal spines of metatibia at first third of metatibial length.

Aedeagus. Fig. 7E–G. Habitus: Fig. 7H.

#### Variation.

Body length: 8.7–9.0 mm, length of elytra: 6.7–7.0 mm, width: 5.4–5.9 mm. Female has small eyes (ratio of diameter/interocular width: 0.6) and antennal club composed of 3 antennomeres being as long as the remaining antennomeres combined.

#### Remarks.

The species is recorded for the first time for China.

### Tetraserica xichouensis sp. n.

Taxon classificationAnimaliaColeopteraScarabaeidae

http://zoobank.org/A19A757A-ED28-4613-B2C0-500F3A6068BF

#### Type material examined.

Holotype: ♂ “China, SE Yunnan, Xichou - E env. 1400–1700m, 13.18.5.95/ 23°22–26'N/ 104°41–49'E L.+R. Businský lgt.” (CPPB).

#### Description.

Body length: 9.0 mm, length of elytra: 6.7 mm, width: 5.8 mm. Surface of labroclypeus and disc of frons glabrous. Smooth area anterior to eye twice as wide as long. Eyes large; ratio of diameter/interocular width: 0.7. Antennal club 1.2 times as long as remaining antennomeres combined. Ratio of length of metepisternum/metacoxa: 1/1.6. Metafemur dull, anterior margin acute, without submarginal serrated line; anterior row of setae-bearing punctures absent; posterior margin with a large sharp hook. Metatibia short and wide, ratio width/length: 1/3.4; basal group of dorsal spines of metatibia at first third of metatibial length.

Aedeagus. Fig. 7I–K. Habitus: Fig. 7L.

Female unknown.

#### Diagnosis.

*Tetraserica xichouensis* sp. n. differs from the very similar *Tetraserica tonkinensis* by the smaller dorsal lobe of the right paramere.

#### Etymology.

The new species is named after its type locality, Xichou.

### Tetraserica mengeana sp. n.

Taxon classificationAnimaliaColeopteraScarabaeidae

http://zoobank.org/63DE72A9-77DD-42CB-AD49-69D2577F3978

#### Type material examined.

Holotype: ♂ [China] “Meng'e, Xishuangbanna, Yunnan, 19.V.1958, 1050–1080m, leg. Hong Chunpei” (IZAS). Paratypes: 1 ♂ [China] “Menghai, Xishuangbanna, Yunnan, 19.VII.1958, 1200–1600m, leg. Wang Shuyong” (IZAS), 1 ♂ [China] “Menghun, Xishuangbanna, Yunnan, 18.V.1958, 1200–1400m, leg. Zhang Yiran” (IZAS), 1 ♂ [China] “Yunnan, Huanglianshan, 2012-V-9, 1800m/ LW-1277” (IZAS), 1 ♂ [China] “Meng'e, Xishuangbanna, Yunnan, 19.V.1958, 1050–1080m, leg. Hong Chunpei” (ZFMK).

#### Description.

**Figure 8.**
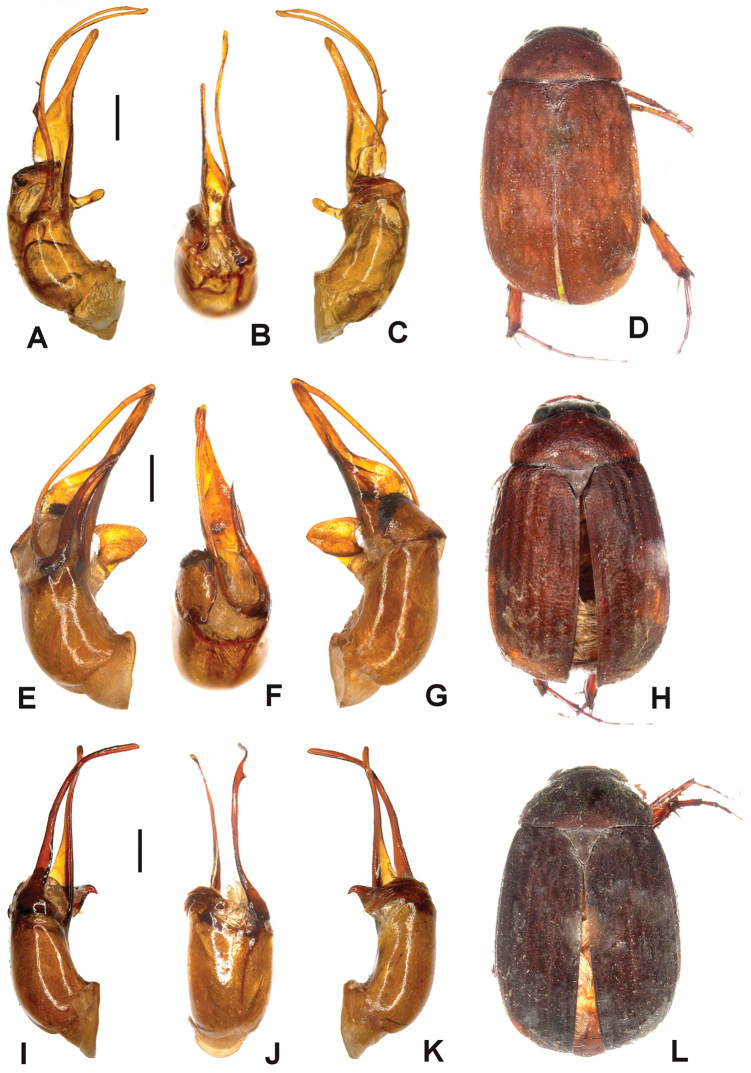
**A–D** *Tetraserica mengeana* sp. n. (holotype) **E–H** *Tetraserica changshouensis* sp. n. (holotype) **I–L** *Tetraserica shangsiensis* sp. n. (holotype). **A, E, I** aedeagus, left side lateral view **C, G, K** aedeagus, right side lateral view **B, F, J** parameres, dorsal view **D, H, L** habitus (not to scale). Scale: 0.5 mm.

Body length: 7.4 mm, length of elytra: 5.7 mm, width: 4.3 mm. Body reddish brown. Surface of labroclypeus and disc of frons glabrous. Smooth area anterior to eye twice as wide as long. Eyes large; ratio of diameter/interocular width: 0.7. Antennal club 1.2 times as long as remaining antennomeres combined. Ratio of length of metepisternum/metacoxa: 1/1.5. Metafemur dull, anterior margin acute, without submarginal serrated line; anterior row of setae-bearing punctures absent; posterior margin straight. Metatibia short and wide, ratio width/length: 1/3.2; basal group of dorsal spines of metatibia at first third of metatibial length. Aedeagus. Fig. 8A–C. Habitus: Fig. 8D.

Female unknown.

#### Variation.

Body length: 7.4–8.6 mm, length of elytra: 5.7–7 mm, width: 4.3–5.9 mm.

#### Diagnosis.

*Tetraserica mengeana* sp. n. differs from all other species with a brush of robust trichome-like spines at the base of the right paramere by the left paramere being split into two filiform branches behind the middle.

#### Etymology.

The new species is named after its type locality, Meng'e.

### Tetraserica changshouensis sp. n.

Taxon classificationAnimaliaColeopteraScarabaeidae

http://zoobank.org/4CBFFD98-D953-43C8-B15B-8B7A32B5666F

#### Type material examined.

Holotype: ♂ [China] “Nanmu Garden, Changshou, Sichuan, 9.VI.1994, 450m, leg. Zhang Youwei” (IZAS). Paratype: 1 ♂ [China] “Mt. Xingdoushan, Lichuan, Hubei, 22.VII.1989, light trap, 810m, leg. Wang Shuyong” (ZFMK).

#### Description.

Body length: 8.8 mm, length of elytra: 6.7 mm, width: 5.3 mm. Surface of labroclypeus and disc of frons glabrous. Smooth area anterior to eye twice as wide as long. Eyes moderately large; ratio of diameter/interocular width: 0.65. Antennal club as long as remaining antennomeres combined. Ratio of length of metepisternum/metacoxa: 1/1.57. Metafemur dull, anterior margin acute, without submarginal serrated line; anterior row of setae-bearing punctures absent; posterior margin straight. Metatibia short and wide, ratio width/length: 1/3.35; basal group of dorsal spines of metatibia at first third of metatibial length.

Aedeagus. Fig. 8E–G. Habitus: Fig. 8H.

Female unknown.

#### Variation.

Body length: 8.0–8.8 mm, length of elytra: 6.0–6.7 mm, width: 5.0–5.3 mm.

#### Diagnosis.

*Tetraserica changshouensis* sp. n. differs from all other species with a brush of robust trichome-like spines at the base of the right paramere by the left paramere being composed of two lobes and the ventral lobe of the right paramere being abruptly and strongly widened at apex.

#### Etymology.

The new species is named after its type locality, Changshou.

### Tetraserica shangsiensis sp. n.

Taxon classificationAnimaliaColeopteraScarabaeidae

http://zoobank.org/6CC6D618-2F79-4442-94C0-B3862420E683

#### Type material examined.

Holotype: ♂ [China] “Hongqi Forestry Farm, Shangsi, Guangxi, 29.V.1999, 300m, leg. Ke Xin” (IZAS). Paratypes: 1 ♂ [China] “Nonggang, Longzhou, Guangxi, 21.V.1982, 240m, leg. Li Fasheng” (CAU), 1 ♂ [China] “Fu'ai, Pingxiang, Guangxi, 17.VI.1976, leg. Zhang Baolin” (ZFMK), 2 ♂♂ [China] “Hong Kong: Lantau I: San Shek Wan; v.1988” (BPBM), 1 ♂ [China] “Hongqi Forestry Farm, Shangsi, Guangxi, 29.V.1999, 300m, leg. Ke Xin” (IZAS), 1 ♂ [China] “Taojiang, Leishan, Guizhou, 5.VII.1988, 1000m, leg. Yang Longlong, light trap” (IZAS).

#### Description.

Body length: 8.1 mm, length of elytra: 6.2 mm, width: 4.6 mm. Surface of labroclypeus and disc of frons glabrous. Smooth area anterior to eye twice as wide as long. Eyes large; ratio of diameter/interocular width: 0.76. Antennal club as long as remaining antennomeres combined. Ratio of length of metepisternum/metacoxa: 1/1.61. Metafemur dull, anterior margin acute, without submarginal serrated line; anterior row of setae-bearing punctures absent; posterior margin straight. Metatibia short and wide, ratio width/length: 1/3.13; basal group of dorsal spines of metatibia at first third of metatibial length.

Aedeagus. Fig. 8I–K. Habitus: Fig. 8L.

Female unknown.

#### Variation.

Body length: 7.0–8.1 mm, length of elytra: 5.6–6.7 mm, width: 4.4–4.8 mm.

#### Diagnosis.

The new species differs from the similar *Tetraserica xichouensis* sp. n. by the left paramere being evenly curved without being clearly bent, and having a tiny lateral tooth before apex which is absent in *Tetraserica xichouensis*.

#### Etymology.

The new species is named after its type locality in Shangsi prefecture (Guangxi province).

### Tetraserica ruiliensis

Taxon classificationAnimaliaColeopteraScarabaeidae

Ahrens, Liu & Fabrizi sp. n.

http://zoobank.org/AA46D770-40D2-4DBE-BC2D-A2C753380157

#### Type material examined.

Holotype: ♂ [China] “Yunnan, Ruili, 2011-IV-27, N: 24.059, E: 97.955, 825m/ LW-1216” (ZFMK).

#### Description.

Body length: 8.1 mm, length of elytra: 5.8 mm, width: 4.8 mm. Surface of labroclypeus and disc of frons glabrous. Smooth area anterior to eye twice as wide as long. Eyes moderately large; ratio of diameter/interocular width: 0.63. Antennal club 1.2 times as long as remaining antennomeres combined. Ratio of length of metepisternum/metacoxa: 1/1.74. Metafemur dull, anterior margin acute, without submarginal serrated line; anterior row of setae-bearing punctures absent; posterior margin straight. Metatibia moderately long and wide, ratio width/length: 1/3.54; basal group of dorsal spines of metatibia at first third of metatibial length.

**Figure 9.**
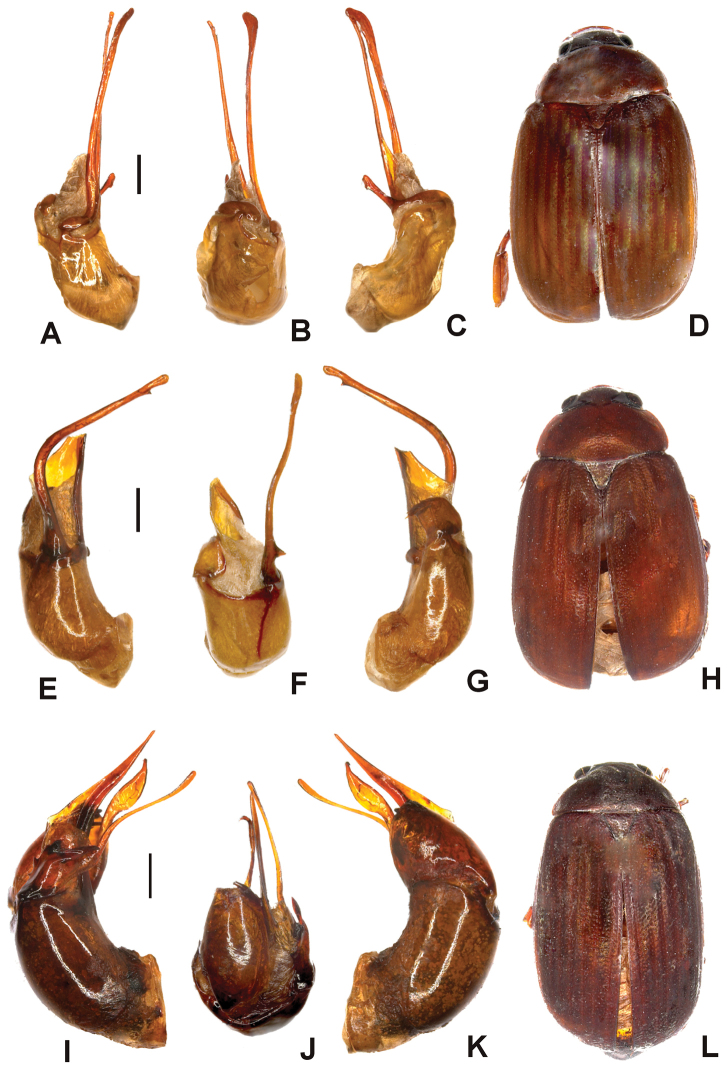
**A–D** *Tetraserica ruiliensis* sp. n. (holotype) **E–H** *Tetraserica linaoshanica* sp. n. (holotype) **I–L** *Tetraserica ruiliana* sp. n. (holotype). **A, E, I** aedeagus, left side lateral view **C, G, K** aedeagus, right side lateral view **B, F, J** parameres, dorsal view **D, H, L** habitus (not to scale). Scale: 0.5 mm.

Aedeagus. Fig. 9A–C. Habitus: Fig. 9D.

Female unknown.

#### Diagnosis.

*Tetraserica ruiliensis* sp. n. differs from all species with straight posterior margin of metafemur, long ventral phallobasis process, and right paramere without brush of spines by the right paramere being simple, not composed of two lobes.

#### Etymology.

The new species is named after its type locality, Ruili.

### Tetraserica linaoshanica sp. n.

Taxon classificationAnimaliaColeopteraScarabaeidae

http://zoobank.org/7B065A6C-2F05-4F13-8433-B399B296CE08

#### Type material examined.

Holotype: ♂ [China] “Mts. Linaoshan, Langping, Tianlin, Guangxi, 28.V.2002, 1400m, leg. Jiang Guofang” (IZAS).

#### Description.

Body length: 8.8 mm, length of elytra: 6.6 mm, width: 5.2 mm. Surface of labroclypeus and disc of frons glabrous. Smooth area anterior to eye twice as wide as long. Eyes moderately large; ratio of diameter/interocular width: 0.63. Antennal club as long as remaining antennomeres combined. Ratio of length of metepisternum/metacoxa: 1/1.5. Metafemur dull, anterior margin acute, without submarginal serrated line; anterior row of setae-bearing punctures absent; posterior margin straight. Metatibia moderately long and wide, ratio width/length: 1/3.69; basal group of dorsal spines of metatibia at first third of metatibial length.

Aedeagus. Fig. 9E–G. Habitus: Fig. 9H.

Female unknown.

#### Diagnosis.

*Tetraserica linaoshanica* sp. n. differs from all *Tetraserica* species with a brush of robust trichome-like spines at the base of the right paramere by the left paramere possessing a small lateral basal tooth.

#### Etymology.

The new species is named after its type locality, Mt. Linaoshan.

### Tetraserica ruiliana sp. n.

Taxon classificationAnimaliaColeopteraScarabaeidae

http://zoobank.org/D5CBA763-DBD0-4B9A-8053-A43F48426C92

#### Type material examined.

Holotype: ♂ [China] “Huyu County, Ruili, Yunnan, 11.VI.1956, 1400m, leg. Zhou Benshou” (IZAS).

#### Description.

Body length: 9 mm, length of elytra: 7 mm, width: 5.3 mm. Surface of labroclypeus and disc of frons glabrous. Smooth area anterior to eye twice as wide as long. Eyes moderately large; ratio of diameter/interocular width: 0.63. Antennal club 1.3 times as long as remaining antennomeres combined. Ratio of length of metepisternum/metacoxa: 1/1.55. Metafemur dull, anterior margin acute, without submarginal serrated line; anterior row of setae-bearing punctures absent; posterior margin straight. Metatibia moderately long and wide, ratio width/length: 1/3.47; basal group of dorsal spines of metatibia at first third of metatibial length.

Aedeagus. Fig. 9I–K. Habitus: Fig. 9L.

Female unknown.

#### Diagnosis.

*Tetraserica ruiliana* sp. n. differs from all *Tetraserica* species with a brush of robust trichome-like spines at the base of the right paramere by the dorsal lobe of the right paramere convexly widened and elongate.

#### Etymology.

The new species is named after its type locality, Ruili.

### Tetraserica anhuaensis sp. n.

Taxon classificationAnimaliaColeopteraScarabaeidae

http://zoobank.org/341DD0C8-BE82-49FD-AA0F-C3666547B313

#### Type material examined.

Holotype: ♂ [China] “Cangchang, Anhua, Hunan, 15.VII.2004, leg. Wang Jiliang” (HBUM). Paratypes: 2 ♂♂ [China] “Hong Kong: N.T. Taipokau 12.VII.1965 Hand Net/ Lee Kit Ming & Hai Wai Ming Malaise Trap Bishop Museum” (BPBM, ZFMK), 1 ♂ [China] “Hong Kong: N.T. Taipokau 27.VI.1964/ M.J. Voss & Wai Ming Hui Collectors Bishop Museum” (BPBM), 1 ♂ [China] “Hong Kong: N.T. Taipokau 20.VI.1964/ Lee Kit Ming & Hai Wai Ming Light Trap Bishop Museum” (BPBM), 1 ♂, 2 ♀♀ [China] “Shekou, Fu‘an, Fujian, 26.VII.1963, leg. Zhang Youwei” (IZAS), 1 ♀ [China] “Shekou, Fu‘an, Fujian, 27.VII.1963, leg. Zhang Youwei” (IZAS), 2 ♂♂ [China] “Mt. Jiulianshan, Longnan, Jiangxi, 6.VI.1975, leg. Zhang Youwei” (IZAS), 1 ♂ [China] “Sidu, Guidong county, Hunan, 10.VII.2008, 774m, leg. Yang Ganyan” (IZAS), 2 ♂♂, 2 ♀♀ [China] “Qiliqiao, Chong'anxingcun, Fujian, 7,12,13.VII.1963, 840m, leg. Zhang Youwei” (IZAS).

#### Description.

Body length: 7.2 mm, length of elytra: 5.2 mm, width: 4.2 mm.

Labroclypeus surface with a few erect setae. Disc of frons with a few single setae. Smooth area in front of eye approximately 4 times as wide as long. Eyes large; ratio of diameter/interocular width: 0.82. Antennal club 1.4 times as long as remaining antennomeres combined.

Ratio of length of metepisternum/metacoxa: 1/1.5. Metafemur with a serrated continuous line beside anterior margin, with fine sparse punctures behind line each bearing a short seta.

Metatibia moderately long, ratio width/length: 1/3.25; basal group of dorsal spines of metatibia behind middle; beside dorsal margin in basal half with a blunt carina being partly finely serrate.

**Figure 10.**
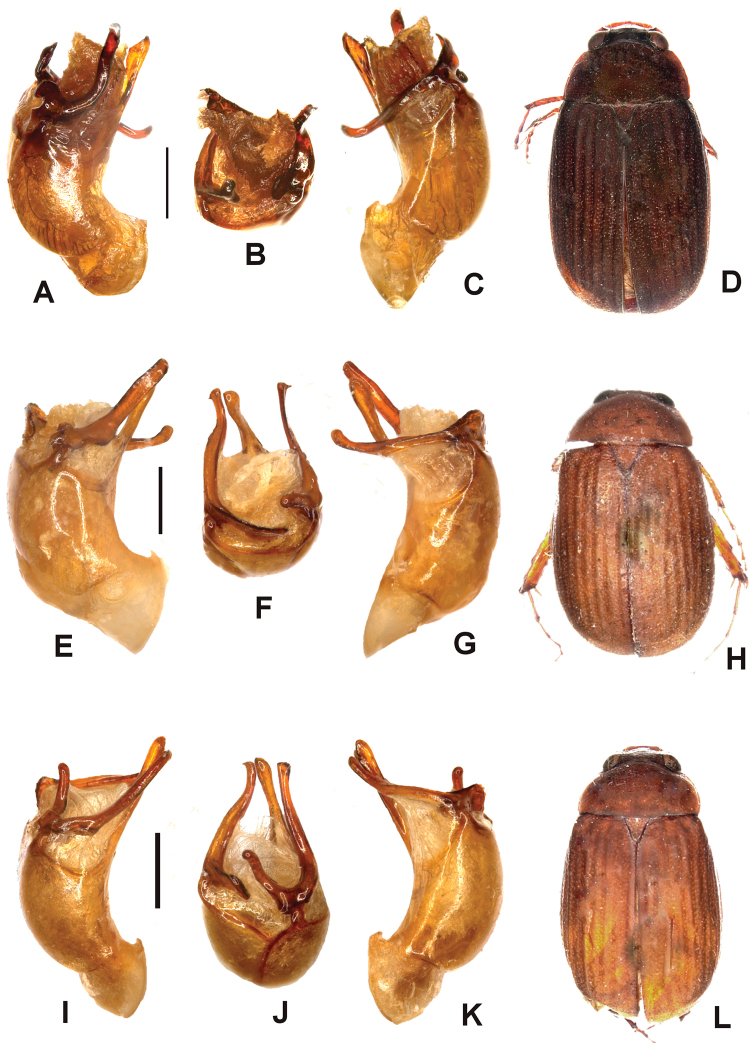
**A–D** *Tetraserica anhuaensis* sp. n. (holotype) **E–H** *Tetraserica leishanica* sp. n. (holotype) **I–L** *Tetraserica yaoanica* sp. n. (holotype). **A, E, I** aedeagus, left side lateral view **C, G, K** aedeagus, right side lateral view **B, F, J** parameres, dorsal view **D, H, L** habitus (not to scale). Scale: 0.5 mm.

Aedeagus. Fig. 10A–C. Habitus: Fig. 10D.

Female unknown.

#### Variation.

Body length: 6.8–7.2 mm, length of elytra: 5.0–5.2 mm, width: 3.8–4.2 mm.

#### Diagnosis.

*Tetraserica anhuaensis* sp. n. differs in a number of characters from all so far known *Tetraserica* species: metafemur possessing a serrated continuous line beside anterior margin and fine sparse punctures with short setae behind line; the labroclypeus and frons having a few erect setae on disc; basal group of dorsal spines situated behind the middle of metatibia; metatibia beside dorsal margin in basal half with a blunt carina being partly finely serrate.

#### Etymology.

The new species is named after its type locality, Anhua.

### Tetraserica leishanica sp. n.

Taxon classificationAnimaliaColeopteraScarabaeidae

http://zoobank.org/E2F9DA66-5DB2-40A9-951B-703742353E08

#### Type material examined.

Holotype: ♂ [China] “Leishan, Guizhou, 28.VI.1988, 800m, leg. Yin Huifen” (IZAS). Paratypes: 1 ♂ [China] “Mts. Fanjingshan, Jiangkou, Guizhou, 13.VIII.1988, 550m, leg. Yang Xingke” (ZFMK), 1 ♂ [China] “Mts. Fanjingshan, Jiangkou, Guizhou, 19.VIII.1988, 550m, leg. Yang Xingke” (IZAS), 1 ♂ [China] “Jiuniutang, Mao‘ershan, Guangxi, 13.VII.1985, 1100m, leg. Liao Subai” (IZAS).

#### Description.

Body length: 6.8 mm, length of elytra: 5 mm, width: 4 mm. Body reddish brown. Labroclypeus surface with a few erect setae. Disc of frons with a few single setae. Smooth area in front of eye approximately 4 times as wide as long. Eyes large; ratio of diameter/interocular width: 0.81. Antennal club 1.4 times as long as remaining antennomeres combined.

Ratio of length of metepisternum/metacoxa: 1/1.68. Metafemur with a serrated continuous line beside anterior margin, with fine sparse punctures behind line each bearing a short seta.

Metatibia moderately long, ratio width/length: 1/3.64; basal group of dorsal spines of metatibia behind middle; beside dorsal margin in basal half with a blunt carina being partly finely serrate.

Aedeagus. Fig. 10E–G. Habitus: Fig. 10H.

Female unknown.

#### Variation.

Body length: 6.8–7.7 mm, length of elytra: 5.0–5.6 mm, width: 4.0–4.6 mm.

#### Diagnosis.

*Tetraserica leishanica* sp. n. is similar to *Tetraserica anhuaensis* externally and in the shape of male genitalia. *Tetraserica leishanica* differs by the reddish brown body colour, more slender metatibia (ratio length/width: >3.6), longer metacoxa. It also differs by the shape of the parameres: the left paramere is straight behind the base (while it is curved in *Tetraserica anhuaensis*), with a long basal lobe (basal lobe in *Tetraserica anhuaensis* is short); the right paramere is more strongly curved externally at apex than in *Tetraserica anhuaensis*.

#### Etymology.

The new species is named after its type locality, Leishan.

### Tetraserica yaoanica sp. n.

Taxon classificationAnimaliaColeopteraScarabaeidae

http://zoobank.org/BF444964-991A-4682-A924-E4761954EE1E

#### Type material examined.

Holotype: ♂ [China] “Yaoan, Lianxian County, Guangdong, 28.VI.1965, leg. Zhang Youwei” (IZAS). Paratypes: **China.** 1 ♂ “Qiliqiao, Chong‘anxingcun, Fujian, 12,13.VII.1963, 840m, leg. Zhang Youwei” (ZFMK), 1 ♂ “Mt. Jiulianshan, Longnan, Jiangxi, 8.VI.1975, light trap, leg. Zhang Youwei” (IZAS), 1 ♀ “Daqiu Forestry Farm, Mt. Jiulianshan, Longnan, Jiangxi, 11.VI.1975, light trap, leg. Zhang Youwei” (IZAS), 1 ♂ “Mt. Jiulianshan, Longnan, Jiangxi, 8.VI.1975, light trap, leg. Zhang Youwei” (IZAS), 1♂ “Changguling Forestry Farm, Mts. Jinggangshan, Jiangxi, 4.VII.1975, light trap” (IZAS), 1 ♂ “Xinzuochang, Boluo, Guangdong, 3.VI.1965, leg. Zhang Youwei” (IZAS), 1 ♂ “Dong'an, Hunan, 20.V.(19)54” (IZAS), 1 ♂ “Guangxi, Shangsi Shiwandashan 2011-VII-7, 263m” (IZAS). **Vietnam.** 3 ♂♂ “Vietnam-N (Na Hang) 160km from Ha Noi, NE env. of Na Hang, 3.–14.6.1996 150-200m leg. A. Napolov & I. Roma” (CNAR, ZFMK), 1 ♂ “S. Vietnam (Cat Tien) 120 km NNE Ho Chi Minh, Cat Tien Nat. Park 30.5.-15.6.1995 leg. A. Napolov” (CNAR).

#### Description.

Body length: 7.2 mm, length of elytra: 5.4 mm, width: 4.3 mm. Body reddish brown. Labroclypeus surface with a few erect setae. Disc of frons with a few single setae. Smooth area in front of eye approximately 4 times as wide as long. Eyes large; ratio of diameter/interocular width: 0.86. Antennal club 1.3 times as long as remaining antennomeres combined.

Ratio of length of metepisternum/metacoxa: 1/1.68. Metafemur with a serrated continuous line beside anterior margin, with fine sparse punctures behind line each bearing a short seta.

Metatibia moderately long, ratio width/length: 1/3.82; basal group of dorsal spines of metatibia behind middle; beside dorsal margin in basal half with a blunt carina being partly finely serrate.

Aedeagus. Fig. 10I–K. Habitus: Fig. 10L.

Female unknown.

#### Variation.

Body length: 6.6–7.9 mm, length of elytra: 4.9–6.0 mm, width: 4.1–4.6 mm. Female has small eyes (ratio of diameter/interocular width: 0.51) and the antennal club composed of 3 antennomeres being as long as the remaining antennomeres combined.

#### Diagnosis.

*Tetraserica yaoanica* sp. n. is very similar to *Tetraserica leishanica* external and in the shape of male genitalia. It differs by the shape of parameres only: basal lobe of the left paramere is short (basal lobe in *Tetraserica leishanica* is long); basal lobe of the right paramere is wide and more than half as long as the paramere (short in *Tetraserica leishanica*).

#### Etymology.

The new species is named after its type locality, Yaoan.

**Figure 11.**
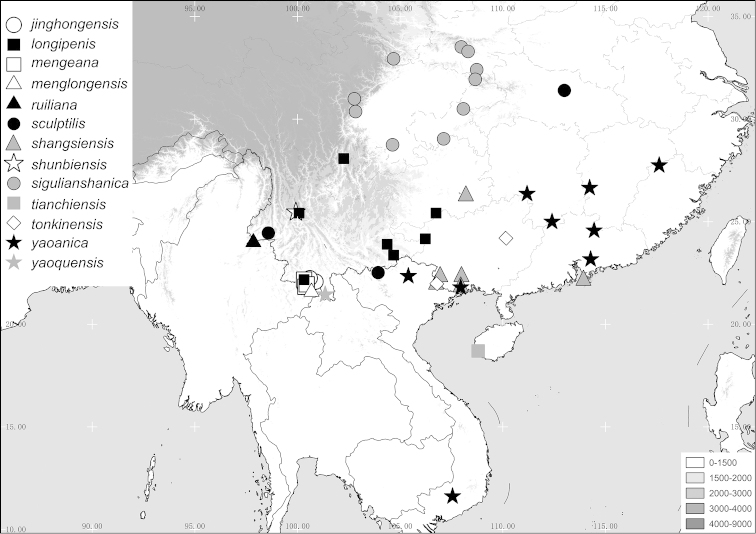
Distribution of *Tetraserica* species: *Tetraserica jinghongensis* sp. n., *Tetraserica longipenis* sp. n., *Tetraserica mengeana* sp. n., *Tetraserica menglongensis* sp. n., *Tetraserica ruiliana* sp. n., *Tetraserica sculptilis* sp. n., *Tetraserica shangsiensis* sp. n., *Tetraserica shunbiensis* sp. n., *Tetraserica sigulianshanica* sp. n., *Tetraserica tianchiensis* sp. n., *Tetraserica tonkinensis* (Moser), *Tetraserica yaoanica* sp. n., and *Tetraserica yaoquensis* sp. n.

**Figure 12.**
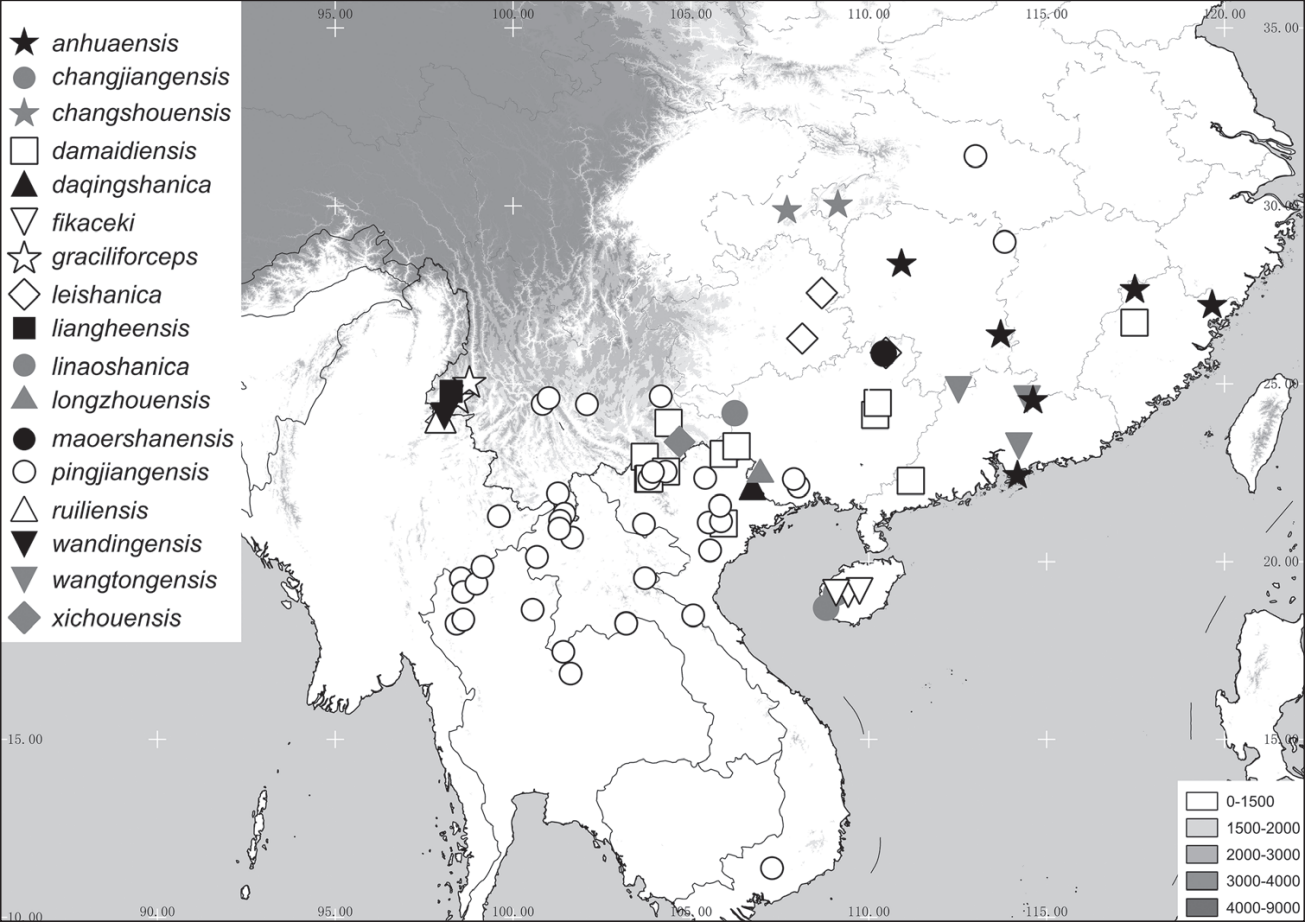
Distribution of *Tetraserica* species: *Tetraserica anhuaensis* sp. n., *Tetraserica changjiangensis* sp. n., *Tetraserica changshouensis* sp. n., *Tetraserica damaidiensis* sp. n., *Tetraserica daqingshanica* sp. n., *Tetraserica fikaceki* sp. n., *Tetraserica graciliforceps* sp. n., *Tetraserica leishanica* sp. n., *Tetraserica liangheensis* sp. n., *Tetraserica linaoshanica* sp. n., *Tetraserica longzhouensis* sp. n., *Tetraserica maoershanensis* sp. n., *Tetraserica pingjiangensis* sp. n., *Tetraserica ruiliensis* sp. n., *Tetraserica wandingensis* sp. n., *Tetraserica wangtongensis* sp. n., and *Tetraserica xichouensis* sp. n.

## Supplementary Material

XML Treatment for Tetraserica

XML Treatment for Tetraserica daqingshanica

XML Treatment for Tetraserica sculptilis

XML Treatment for Tetraserica wangtongensis

XML Treatment for Tetraserica maoershanensis

XML Treatment for Tetraserica fikaceki

XML Treatment for Tetraserica changjiangensis

XML Treatment for Tetraserica sigulianshanica

XML Treatment for Tetraserica damaidiensis

XML Treatment for Tetraserica shunbiensis

XML Treatment for Tetraserica longzhouensis

XML Treatment for Tetraserica yaoquensis

XML Treatment for Tetraserica longipenis

XML Treatment for Tetraserica jinghongensis

XML Treatment for Tetraserica menglongensis

XML Treatment for Tetraserica tianchiensis

XML Treatment for Tetraserica liangheensis

XML Treatment for Tetraserica graciliforceps

XML Treatment for Tetraserica pingjiangensis

XML Treatment for Tetraserica wandingensis

XML Treatment for Tetraserica tonkinensis

XML Treatment for Tetraserica xichouensis

XML Treatment for Tetraserica mengeana

XML Treatment for Tetraserica changshouensis

XML Treatment for Tetraserica shangsiensis

XML Treatment for Tetraserica ruiliensis

XML Treatment for Tetraserica linaoshanica

XML Treatment for Tetraserica ruiliana

XML Treatment for Tetraserica anhuaensis

XML Treatment for Tetraserica leishanica

XML Treatment for Tetraserica yaoanica
